# Vitamin A, Vitamin D, Iron, and Zinc in Relation to Anemia Risk: Observational Evidence and Mendelian Randomization

**DOI:** 10.3390/nu17203220

**Published:** 2025-10-14

**Authors:** Jiapeng Tang, Yaqing Tan, Yanhua Chen, Fei Wang, Tingting Wang, Mengting Sun, Manjun Luo, Ye Chen, Yuting Wen, Zhanwen Li, Kebin Chen, Kaiwei Luo, Jiabi Qin

**Affiliations:** 1 Department of Epidemiology and Health Statistics, Xiangya School of Public Health, Central South University, Changsha 410013, China; 256902017@csu.edu.cn (J.T.); wangting123@csu.edu.cn (T.W.); 226901004@csu.edu.cn (M.S.); luomj0810@csu.edu.cn (M.L.); 236912089@csu.edu.cn (Y.C.); wenyuting08@csu.edu.cn (Y.W.); 246911014@csu.edu.cn (Z.L.); 246907001@csu.edu.cn (K.C.); 2 Hunan Provincial Center for Disease Control and Prevention, Changsha 410153, China; yaqing998@163.com (Y.T.); hncdccyh@163.com (Y.C.); cdc.wangfei@hotmail.com (F.W.); 3 Department of Epidemiology and Health Statistics, School of Public Health, Kunming Medical University, Kunming 650500, China; 4 Yunnan Key Laboratory of Cross-Border Infectious Disease Prevention and New Drug Development, Kunming 650500, China; 5 Yunnan Provincial Key Laboratory of Public Health and Biosafety, Kunming 650500, China

**Keywords:** anemia, influencing factors, micronutrients, Mendelian randomization

## Abstract

**Background**: Anemia remains an important public health problem worldwide. Investigating the potential influencing factors of anemia can provide a reference for improving anemia status. This study aimed to identify factors influencing anemia in school-age children and assess associations/causal relationships between micronutrients (vitamin A, vitamin D, iron, and zinc) and anemia risk. **Methods**: This study included 1725 school-age children. Factors associated with anemia were identified using multivariable-adjusted logistic regression. Associations of serum micronutrients with anemia were analyzed, and non-linear relationships were examined. Causality was assessed using two-sample Mendelian randomization (MR) analysis. **Results**: Daytime outdoor activity, milk consumption, school location, picky eating, and serum ferritin deficiency were associated with anemia (*p* < 0.05). Higher serum vitamin A (Q4 vs. Q1: *OR* = 0.548; *P*_trend_ = 0.027) and higher serum ferritin (Q4 vs. Q1: *OR* = 0.470; Q3 vs. Q1: *OR* = 0.609; *P*_trend_ = 0.011) were inversely associated with anemia. RCS indicated a J-shaped non-linear relationship between serum ferritin and anemia risk. MR analysis showed that serum 25-hydroxyvitamin D (*OR* = 0.864, 95%*CI*: 0.757–0.986, *p* = 0.030), serum ferritin (*OR* = 0.656, 95%*CI*: 0.588–0.731, *p* < 0.001), and serum iron (*OR* = 0.793, 95%*CI*: 0.681–0.925, *p* = 0.003) significantly reduced anemia risk with the IVW method. Sensitivity analyses showed no heterogeneity, pleiotropy, or reverse causality. **Conclusions**: This study found that daytime outdoor activity time, weekly milk consumption frequency, school location, picky eating, and serum ferritin deficiency are closely associated with anemia in school-aged children. Additionally, serum vitamin A, vitamin D, serum iron, and serum ferritin levels are also linked to anemia. These findings collectively highlight the importance of lifestyle factors and specific micronutrients in influencing anemia among school-aged children, providing valuable insights for targeted prevention and intervention strategies. Future intervention trials focusing on these key factors could further validate their practical application value.

## 1. Introduction

Anemia is a common clinical condition in which the volume of red blood cells in human peripheral blood is reduced below the lower limit of the normal range. Anemia remains an important public health problem worldwide, and it is strongly associated with increased morbidity and mortality resulting from adverse health outcomes across populations. According to the latest Burden of Disease study, the global prevalence of anemia in 2021 was 24.3%, affecting more than 1.9 billion people and resulting in 52 million years lost due to disability (YLDs) [1]. During the same period, the number of people suffering from anemia in China is 136 million, with a prevalence rate of 8.9% [2]. The World Health Organization (WHO) reported that the global prevalence of anemia among students was 25.4%, which is second only to children aged 6–59 months and women of childbearing age [3]. A meta-analysis study showed that the prevalence of anemia among school-age children in Latin America and the Caribbean was 17.49% [4]. Data from the 2016–2017 China Nutrition and Health Surveillance revealed an overall anemia prevalence of 4.4% among Chinese elementary school students. A significantly higher prevalence rate was observed in rural areas (4.99%) compared to urban areas [5]. The consequences of anemia in students are substantial, including growth retardation, increased susceptibility to infections, and impaired cognitive and motor development [6,7]. Furthermore, long-term effects may include reduced social engagement and low learning or working efficiency [8]. It is evident that anemia not only affects the health and quality of life of individuals but may also have a negative impact on the economic development of societies and countries.

There are various factors affecting anemia in school-age children, including demographic and socioeconomic factors, environmental factors, and genetic factors. Genetic factors, primarily associated with specific hemoglobinopathies such as thalassemia and sickle cell anemia, are generally less amenable to modification through conventional interventions [9]. Among demographic and socioeconomic factors, gender, ethnicity, and nutritional status are closely related to the occurrence of anemia [5,10,11]. Among environmental factors, in addition to natural environmental factors, school and family environment are also important influencing factors [12,13]. While lots of research exists on anemia risk factors in school-age children, most of these studies lack comprehensiveness in examining such factors. Furthermore, with the increasing number of population-based studies and animal experiments investigating the association between micronutrients (such as vitamin A, vitamin D, iron, and zinc) and anemia, coupled with their potential physiological linkages, the impact of these micronutrients on anemia has garnered significant attention. The latest meta-analysis found that vitamin D deficiency is associated with multiple noncommunicable diseases, including anemia [14]. This finding is consistent with the results of a meta-analysis of observational studies [15]. However, another meta-analysis of intervention studies suggests that vitamin D supplementation does not significantly elevate hemoglobin (Hb) concentrations [16]. Existing evidence indicates that the impact of vitamin D on anemia may be closely related to its role in regulating erythropoiesis and iron metabolism [17,18,19]. A meta-analysis of clinical trials demonstrated that vitamin A supplementation reduced the risk of anemia by 26% and increased hemoglobin levels compared to untreated groups [20]. Current evidence indicates that the role of vitamin A in anemia pathogenesis is primarily associated with the regulation of erythropoiesis, modulation of iron metabolism, and enhancement of immune function to mitigate the risk of infection [21,22,23,24]. In addition, studies have also focused on the association of human iron and zinc levels with anemia, but the findings are not entirely consistent [25,26,27,28]. In addition, the number of studies on the dose–response relationship between the above micronutrients and anemia is limited, with only two studies using restricted cubic splines (RCS) to explore the dose–response relationship between serum vitamin D levels and anemia, as well as iron deficiency anemia (IDA) in American adults [29,30]. No dose–response studies of vitamin A, iron, and zinc in relation to anemia have been conducted.

Furthermore, beyond utilizing observational study data to explore the association between common micronutrients and disease, Mendelian randomization (MR) studies can also provide reliable evidence of causal associations. The primary strength of MR lies in its use of genetic variants, assigned randomly at conception, as instrumental variables (IVs) for exposures. This design inherently mitigates reverse causation, as genotypes precede disease outcomes. Furthermore, by utilizing genetically predicted exposure levels, MR reduces susceptibility to confounding from acquired environmental, behavioral, and lifestyle confounders, thereby closely approximating the causal inference framework of randomized controlled trials [31,32]. While MR has been extensively applied to investigate causal effects of micronutrients on various health outcomes [33,34], studies specifically examining the causal roles of common micronutrients (vitamin A, vitamin D, iron, and zinc) in anemia etiology remain limited [29,35,36,37]. Of note, with the continuous updating of genome-wide association study (GWAS) databases, GWAS data from larger samples can provide more reliable evidence for the causal associations between the above common micronutrients and anemia.

This study aimed to comprehensively investigate factors of anemia among school-age students and evaluate the associations between serum levels of vitamin A, vitamin D, iron, and zinc and anemia risk, utilizing nutritional health surveillance data from Hunan Province, China. Furthermore, a two-sample MR study was conducted to further investigate the causal associations between the above four micronutrients and anemia. This study will provide a scientific basis for targeted improvement of anemia in primary and secondary school students.

## 2. Methods

### 2.1. Cross-Sectional Study

#### 2.1.1. Study Design and Participants

This study employed a cross-sectional design. Data were derived from the 2021 and 2023 Student Nutrition and Health Status Monitoring and Evaluation Project of Hunan Provincial. Spearheaded by the Chinese Center for Disease Control and Prevention, this project aimed to assess the nutritional health status of students in areas implementing the Nutrition Improvement Programme for Rural Compulsory Education Students (NIPRCES). A multistage stratified cluster random sampling method was used to select 4 counties from the 37 national pilot counties implementing NIPRCES in Hunan Province as the key monitoring counties. Each key monitoring county then selected eight representative schools as the key monitoring schools, based on the location of the school and meal provision models. Within each selected school, students were stratified by grade level, with approximately 40 students randomly sampled per grade to ensure balanced gender representation. Therefore, the monitoring data are representative of the main study population in this study, primary and secondary school students aged 6–17 in the areas implementing NIPRCES of Hunan Province.

After excluding participants with incomplete questionnaires, biological indicator values below detection limits, missing physical examination data, age outside the 6–17 year range, or duplicate measurements, 1725 participants were finally included in this study for analysis. The study was approved by the Ethics Committee of the Chinese Center for Disease Control and Prevention (Approval No. 2021-018). All participants and their guardians provided written informed consent after being fully informed.

#### 2.1.2. Questionnaire Survey and Sample Collection

The questionnaires and survey forms used in this study were developed by nutrition experts organized by the Institute of Nutrition of the Chinese Center for Disease Control and Prevention. Their validity was confirmed through expert panel discussions and pilot testing. The following influencing factors were included in this study: (1) The basic information of the students (gender, ethnicity, school segments, age, and left-behind children) and exercise and dietary behaviors (daytime outdoor activity time, daily moderate to vigorous physical activity time, daily meal frequency, picky eating, and frequency of various types of food consumed per week). The above information was self-reported by students. (2) The basic school information (school location, daily meal frequency provided by the school, whether the school maintains student health records, whether the school has a cafeteria, and health education) was obtained from the principal in charge of the school. (3) The county-level variables (terrain and altitude) were obtained from the head of the Education Bureau. In addition, the physical examinations were conducted according to the testing methods and instruments specified in the National Survey on Student Physical Fitness and Health. Height and weight were measured in the early morning on an empty stomach. Height was measured using a mechanical height-sit-height meter, measured in centimeters (cm) with an accuracy of 0.1 cm. Weight was measured using an electronic weighing scale or lever scale, measured in kilograms (kg) with an accuracy of 0.1 kg. Body mass index (BMI) was calculated as weight (kg) divided by height (m) squared. The nutritional status of children can be divided into growth retardation, underweight, normal weight, overweight, and obesity according to the Chinese screening standards for malnutrition of school-age children and adolescents (WS/T456-2014) [38] and the Chinese screening standards for malnutrition of school-age children and adolescents (WS/T586-2018) [39].

Peripheral blood samples were collected from students by professional nurses in the morning after at least 10 h of fasting and hemoglobin was detected by cyanide methemoglobin test using a hemoglobin analyzer (HemoCue AB, Ängelholm, Sweden, HemoCue201+). In addition, 5 mL of fasting elbow venous blood was also collected from students in the morning by professional nurses. The blood samples were left to stand at room temperature (22–25 °C) for 20–30 min; then, the blood samples were centrifuged at 3500 r/min for 15 min within two hours. The upper serum layer was aspirated and transferred into serum freezing tubes of 1–1.5 mL each, which were stored in freezing cartridges for subsequent testing. Serum retinol and serum 25-hydroxyvitamin D were detected by high-performance liquid chromatography–tandem mass spectrometry. Serum ferritin was detected by electrochemiluminescence, and serum zinc was detected by mass spectrometry.

#### 2.1.3. Exposure and Outcome

The exposure factors of interest in this study were levels of four common micronutrients (vitamin A, vitamin D, iron, and zinc). In the analysis of the influencing factors, vitamin A status was assessed using the criteria of vitamin A deficiency screening (WS/T553-2017): serum retinol < 0.2 μg/mL indicated deficiency, 0.2–0.3 μg/mL subclinical deficiency, and ≥0.3 μg/mL normal [40]. Due to low prevalence, deficiency and subclinical deficiency were combined as “vitamin A deficiency” in this study. Vitamin D status was assessed using the screening standard for vitamin D deficiency (WS/T667-2020): serum 25-hydroxyvitamin D < 12 ng/mL indicated deficiency, 12–20 ng/mL insufficient, and ≥20 ng/mL normal [41]. Iron status was assessed using WHO guidelines for assessing iron status: serum ferritin < 15 μg/L indicated deficiency and ≥15 ng/mL normal [42]. For zinc status, where standardized criteria are lacking, deficiency was operationally defined as concentrations below the 10th percentile of the present study (serum zinc < 4.80 µmol/L). In the association analysis with anemia, the four micronutrient categories were used to equate the participants into four groups (Q1, Q2, Q3, and Q4) according to their respective P25, P50, and P75, with the first quartile (Q1 group) as the reference category.

The outcome of interest in this study was anemia, defined as a decrease in peripheral blood erythrocyte volume, with reference to the WHO diagnostic criteria for anemia Hb levels below the normal cut-off point. The Hb were first adjusted based on the altitude of each monitoring point, and then anemia was diagnosed based on the WHO diagnostic criteria for anemia, which was diagnosed as Hb < 115 g/L in children aged 5–11 years, Hb < 120 g/L in children aged 12–14 years, Hb < 130 g/L in boys aged 15–18 years, and Hb < 120 g/L in girls (non-pregnant women) aged 15–18 years [43].

#### 2.1.4. Statistical Analysis of Cross-Sectional Study

All statistical analyses were performed using SPSS (version 26.0) software and R (version 4.2.3) software. In the description of the basic characteristics of the participants, categorical variables were described in the form of a number (proportion), while continuous variables that are approximately normally distributed were described in the form of mean ± standard deviation. Intergroup comparisons were performed using *χ*^2^ tests for categorical variables and a *t*-test for continuous variables. The prevalence of anemia and its 95% confidence intervals (95%*CIs*) were calculated based on an approximation of the binomial distribution to the normal distribution. Variables identified as significant (*p* < 0.05) in univariable logistic regression analyses were subsequently assessed for multicollinearity using tolerance and variance inflation factor (*VIF*) diagnostics. After excluding collinear variables (*VIF* > 10 or tolerance < 0.01), multivariable logistic regression with forward selection was performed to identify factors of anemia. Odds ratio (*OR*) with its 95%*CI* was used for risk assessment. The logistic regression model was used to estimate the *OR* and 95%*CI*s between the four common micronutrients (vitamin A, vitamin D, iron, and zinc) and anemia. Three models were constructed for the above analysis. Model 1 was unadjusted for any covariates. Model 2 was adjusted for basic information, including sex, ethnicity, school segments, age, nutritional status, and left-behind children. Model 3 was further adjusted for other common micronutrients (serum vitamin A, serum vitamin D, serum ferritin, and serum zinc) except for the grouping variables based on Model 2. To avoid overfitting in Model 3 due to multicollinearity among the four micronutrients, tolerance and *VIF* were also employed to test for multicollinearity among these micronutrients. Tests for linear trends were performed by using the quartile median value as a quasi-continuous variable in the models. RCS regression was conducted to address the potential non-linearity of the association between the four common micronutrients (vitamin A, vitamin D, iron, and zinc) and anemia. Subgroup analyses were conducted to further evaluate the robustness of the significant associations between common micronutrients and risks of anemia. Two-sided tests were used for this study and a *p*-value < 0.05 was considered statistically significant unless otherwise stated.

### 2.2. MR Analysis

#### 2.2.1. Study Design and Data Sources

Three core assumptions need to be met when performing MR analysis: (1) correlation assumption: there is a strong correlation between IVs and common micronutrient levels (vitamin A, vitamin D, iron, and zinc); (2) independence assumption: IVs cannot be associated with confounding factors; (3) exclusivity assumption: IVs cannot directly cause anemia but can only indirectly affect the occurrence of anemia through the sole pathway of common micronutrient levels (vitamin A, vitamin D, iron, and zinc). Figure S1 shows the overall design of the present study, which employs MR analysis using a two-sample approach to investigate the causal relationship between common micronutrient levels (vitamin A, vitamin D, iron, and zinc) and anemia.

The basic information on the GWAS data for common micronutrients (vitamin A, vitamin D, iron, and zinc) and anemia used in this study is summarized in Table S1, all of which are publicly accessible and all of which were obtained from the original studies with informed consent and ethical approval from the patients. The GWAS data for serum retinol were obtained from an inverse variance weighted GWAS meta-analysis study conducted by Reay WR et al. [44], which included the results of two cohort studies (INTERVAL and METSIM cohorts), with a combined total of 17,268 European participants, and the data contained 8,173,975 single-nucleotide polymorphisms (SNPs). The GWAS data for serum 25-hydroxyvitamin D was obtained from the GWAS analysis conducted by Revez JA et al. [45], which included a total of 417,580 European subjects and contained 880,678 SNPs. The GWAS data for erythrocyte zinc was obtained from the GWAS of blood copper, selenium, and zinc levels by Evans DM et al. [46]. The zinc dataset comprised 2603 Australian participants from the Queensland Institute of Medical Research cohort, genotyped at 2,540,325 SNPs. The GWAS summary statistics for four serum iron biomarkers (serum ferritin, serum iron, total iron-binding capacity, and transferrin saturation) were sourced from a meta-analysis integrating GWAS data from the deCODE Genetics study in Iceland, the INTERVAL study in the UK, and The Danish Blood Donor Study by Bell S et al. [47]. The analysis included European participants with the following sample sizes: serum ferritin (n = 246,139), serum iron (n = 163,511), total iron-binding capacity (n = 135,430), and transferrin saturation (n = 131,471). The GWAS summary statistics for anemia were derived from the FinnGen consortium’s accessible GWAS datasets in 2024, and the study population consisted of 33,700 anemia cases and 104,642 controls with anemia diagnoses that met WHO criteria [48]. The FinnGen study is a large-scale genomics initiative that has analyzed more than 500,000 Finnish biobank samples and correlated genetic variants with health data to understand disease mechanisms and susceptibility.

#### 2.2.2. Instrumental Variables

In order to meet the three core assumptions, the IVs in the present study needed to fulfill the following requirements: (1) significant correlation with the exposure phenotype (*p* < 5 × 10^−8^) and *F*-statistics of >10 for each IV; (2) chain disequilibrium analysis of the SNPs, with the specific parameter set as physical distance = 10,000 kb and *r*^2^ < 0.001; (3) minor allele frequencies (MAF) > 0.01; (4) nonpalindromic SNPs (when palindromic SNPs existed, harmonization to exclude palindromic and incompatible SNPs); and (5) eliminate the IVs that were strongly associated with the outcome (*p* < 5 × 10^−8^).

#### 2.2.3. Statistical Analysis of MR Analysis

The workflow of the present study is presented in Figure S2. In the MR analyses, inverse variance weighted (IVW) method was used to explore the association between common micronutrient levels (vitamin A, vitamin D, iron, and zinc) and anemia as the primary method. Estimates from other methods, including MR-Egger method, weighted median method, weighted mode method, and simple mode method, were used as sensitivity analyses. A causal association can be considered to exist when the results of the IVW model show the significant presence of causal effects and the direction of the effect estimates of the other four types of models is consistent with the IVW model. The Cochran’s Q test and MR-Egger intercept test were used, respectively, to detect heterogeneity and horizontal directional pleiotropy. We used the MR-Pleiotropy Residual Sum and Outlier methods (MR-PRESSO) tests to remove outliers to correct for horizontal multiplicity. In addition, leave-one-out analyses were performed to assess whether MR estimates were driven or biased by single SNPs. To further exclude the influence of potential confounders, the LDTrait tool (https://ldlink.nih.gov/?tab=home (accessed on 26 February 2025)) was used to query all potentially relevant phenotypes of the IVs. Finally, this study used reverse MR analysis to clarify the direction of causal associations in order to exclude the interference of reverse causal associations. In the above analyses, *OR* and its 95%*CIs* were used to assess the strength of causality between common micronutrients levels (vitamin A, vitamin D, iron, and zinc) and anemia. All the analyses were conducted by using R (version 4.2.3) software. Two-sided tests were used for this study and a *p*-value < 0.05 was considered statistically significant unless otherwise stated.

## 3. Results

### 3.1. Characteristics of Participants

A total of 1725 students aged 6–17 years were included in this study. In the total study population, the prevalence of anemia was 8.99% (95%*CI*: 7.63–10.34), as shown in Table S2. The characteristics of the population classified by anemia status are shown in Table 1. There were no significant differences (*p* < 0.05) between anemic and non-anemic students in terms of gender, ethnicity, school segments, age, nutritional status, left-behind children, serum vitamin A level, serum vitamin D level, and serum zinc level, but serum ferritin level was significantly higher in non-anemic students than in anemic students (69.26 ± 49.85 µg/L vs. 58.70 ± 43.78 µg/L, *p* = 0.011).

### 3.2. Logistic Regression Model for Risk Factors Associated with Anemia

As shown in Table S3, the results of univariable logistic regression analysis showed that students who had daytime outdoor activity time for 30–59 min (*OR* = 0.379, 95%*CI*: 0.231–0.622, *p* < 0.001) and ≥60 min (*OR* = 0.540, 95%*CI*: 0.344–0.849, *p* = 0.008) compared to <30 min, weekly milk consumption frequency of not daily (*OR* = 0.649, 95%*CI*: 0.421–0.999, *p* = 0.050) and daily (*OR* = 0.428, 95%*CI*: 0.257–0.712, *p* = 0.001) compared to barely drink, and weekly fruits consumption frequency of daily (*OR* = 0.492, 95%*CI*: 0.243–0.995, *p* = 0.048) compared to barely eat had a lower risk of anemia. However, students whose school location was rural towns/villages (*OR* = 2.157, 95%*CI*: 1.537–3.029, *p* < 0.001) compared to county town, who were picky eaters (*OR* = 1.658, 95%*CI*: 1.190–2.308, *p* = 0.003), and had serum vitamin A deficiency (*OR* = 1.642, 95%*CI*: 1.090–2.475, *p* = 0.018) and serum ferritin deficiency (*OR* = 4.908, 95%*CI*: 2.821–8.539, *p* < 0.001) compared to normal had a higher risk of anemia.

**Figure 1:**
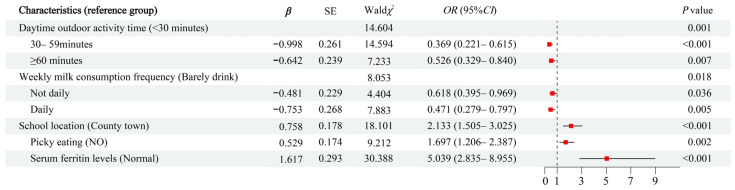
Multivariate logistic regression model for risk factors associated with anemia. Abbreviations: *OR*, odds ratio; *CI*, confidence interval.

A multicollinearity test of the seven variables with statistically significant differences in the univariate analysis revealed that all seven factors had a tolerance greater than 0.1 and a *VIF* less than 10, indicating no significant multicollinearity (details in Table S4). A multivariate logistic regression analysis was performed using forward stepwise regression and the results are shown in Figure 1. The results showed that students who had daytime outdoor activity time for 30–59 min (*OR* = 0.369, 95%*CI*: 0.221–0.615, *p* < 0.001) and ≥60 min (*OR* = 0.526, 95%*CI*: 0.329–0.840, *p* = 0.007) compared to <30 min and weekly milk consumption frequency of not daily (*OR* = 0.618, 95%*CI*: 0.395–0.969, *p* = 0.036) and daily (*OR* = 0.471, 95%*CI*: 0.279–0.797, *p* = 0.005) compared to barely drink had a lower risk of anemia. However, students whose school location was rural towns/villages (*OR* = 2.133, 95%*CI*: 1.505–3.025, *p* < 0.001) compared to county town, who were picky eaters (*OR* = 1.697, 95%*CI*: 1.206–2.387, *p* = 0.002), and had serum ferritin deficiency (*OR* = 5.039, 95%*CI*: 2.835–8.955, *p* < 0.001) compared to normal had a higher risk of anemia.

### 3.3. Associations Between Common Micronutrient Levels and the Risk of Anemia

**Figure 2:**
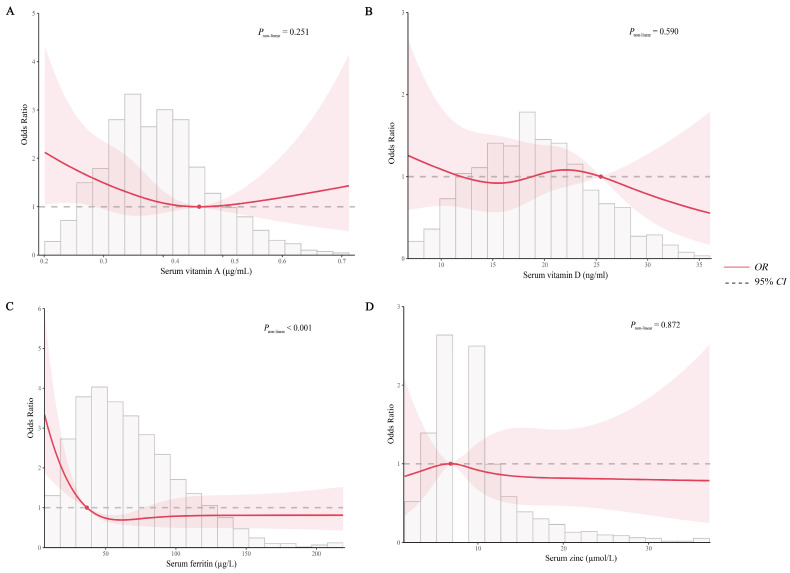
Restricted cubic splines-based modeling for the associations between common micronutrient levels and the risk of anemia. (**A**) Serum vitamin A; (**B**) Serum vitamin D; (**C**) Serum ferritin; (**D**) Serum zinc. The model was adjusted for sex, ethnicity, school segments, age, nutritional status, and left-behind children and common micronutrient quartiles (serum vitamin A, serum vitamin D, serum ferritin, and serum zinc) except for the grouping variables. Abbreviations: *OR*, odds ratio; *CI*, confidence interval.

Table 2 presents associations between four micronutrients and anemia. The results of the multicollinearity test indicated that the four micronutrients had a tolerance greater than 0.1 and a *VIF* less than 10, indicating no significant multicollinearity. For serum vitamin A, no significant association was found between serum vitamin A levels and anemia in any of the three models. However, after dividing serum vitamin A levels into four groups according to quartiles, it was found that, in all three models, the risk of anemia was significantly lower in group Q4 (≥0.44 μg/mL) compared to group Q1 (<0.33 μg/mL), and the results of the linear trend test were statistically significant (*p* < 0.05). After adjusting for sex, ethnicity, school segments, age, nutritional status, left-behind children, serum vitamin D, serum zinc, and serum ferritin levels in Model 3, the risk of anemia was significantly lower in the Q4 group (*OR* = 0.548, 95%*CI*: 0.332–0.903, *p* = 0.018) compared with the Q1 group. For serum ferritin, a significant association between serum ferritin levels and anemia was found in all three models (*p* < 0.05), and the risk of anemia was significantly lower in group Q3 (61.03–88.43 µg/L) and Q4 (≥88.43 µg/L) compared to group Q1 (<38.90 µg/L) with a statistically significant linear trend (*p* < 0.05). Of note, the risk of anemia was also significantly lower in group Q2 (38.90–61.03 µg/L) compared to group Q1 (<38.90 µg/L) in Model 1 (*p* = 0.025). After adjusting for sex, ethnicity, school segments, age, nutritional status, left-behind children, serum vitamin D, serum zinc, and serum vitamin A levels in Model 3, the risk of anemia was significantly lower in the Q3 group (*OR* = 0.609, 95%*CI*: 0.386–0.961, *p* = 0.033) and Q4 group (*OR* = 0.470, 95%*CI*: 0.287–0.772, *p* = 0.003) compared with the Q1 group. No significant associations were observed for serum vitamin D or zinc as continuous or grouped variables in any model (*p* > 0.05). Further analysis with multivariable-adjusted RCS revealed that there was no statistically significant non-linear relationship between serum vitamin A, vitamin D, and zinc levels and anemia risk (*P*_non-liner_ > 0.05). However, the non-linear trend test for serum ferritin and anemia was statistically significant (*P*_non-liner_ < 0.001). The RCS revealed a significant J-shaped non-linear relationship between serum ferritin and anemia risk with a cut-off value of 36.696 µg/L. The detailed results are presented in Figure 2.

### 3.4. Subgroup Analysis

To further evaluate the robustness of the results, the present study explored the association of serum vitamin A quartile and serum ferritin quartile with anemia in different subgroups of the population by using the covariates in Model 3 as subgroup variables. Subgroup analyses demonstrated that the association between serum vitamin A quartile and serum ferritin quartile and the risks of anemia remained largely consistent across subgroups, indicating stable and reliable findings. The detailed results are presented in Tables S5 and S6.

### 3.5. Causal Relationship Between Serum Iron Status Indicators and Anemia

IVW analysis revealed a negative causal association between serum ferritin (*OR* = 0.643, 95%*CI*: 0.570–0.726, *p* < 0.001), serum iron (*OR* = 0.821, 95%*CI*: 0.688–0.980, *p* = 0.029), and anemia risk (Table 3). However, there were no significant causal associations between total iron-binding capacity (*OR* = 1.207, 95%*CI*: 0.961–1.515, *p* = 0.106), transferrin saturation (*OR* = 0.891, 95%*CI*: 0.776–1.024, *p* = 0.103), and anemia risk. Heterogeneity was observed by Cochran Q test (*p* < 0.05) in all four serum iron status indicators (Table 4). To exclude significant heterogeneity caused by outliers, MR-PRESSO was used to identify potential outliers. The results indicated that one SNP (rs17476364) in serum ferritin, two SNPs (rs4774514 and rs7385804) in serum iron, two SNPs (rs1894692 and rs199138) in total iron-binding capacity, and two SNPs (rs4774514 and rs855791) in transferrin saturation were potential outliers.

After removing the outlier and re-performing MR analysis, IVW analysis revealed a negative causal association between serum ferritin (*OR* = 0.656, 95%*CI*: 0.588–0.731, *p* < 0.001), serum iron (*OR* = 0.793, 95%*CI*: 0.681–0.925, *p* = 0.003), and anemia risk, which were consistent with the results before outlier removal (Table 3). The other four MR models all provided causal effect estimates consistent in direction with the IVW model. Also, there were still no significant causal associations between total iron-binding capacity (*OR* = 1.071, 95%*CI*: 0.897–1.280, *p* = 0.447), transferrin saturation (*OR* = 1.008, 95%*CI*: 0.860–1.181, *p* = 0.920), and anemia risk after removing the outliers. After removing the outliers, the MR-Egger intercept indicated no significant horizontal pleiotropy (*p* > 0.05) and no heterogeneity was observed by the Cochran Q test in all four serum iron status indicators (Table 4). The scatter plot of the causal association between serum iron status indicators and anemia is shown in Figures S3 and S4. The leave-one-out sensitivity analysis demonstrated the robustness of the results (Figures S5 and S6). In addition, we did not find possible confounded SNPs by using LDTrait tool. A reverse MR analysis of serum ferritin, serum iron, and anemia was performed. None of the five models found the presence of causal associations (*p* > 0.05), excluding the possibility of reverse causal association (Table 5).

### 3.6. Causal Relationship Between Serum 25-Hydroxyvitamin D and Anemia

First, there was no statistically significant evidence of a potential causal effect of serum 25-hydroxyvitamin D on anemia (*OR* = 0.918, 95%*CI*: 0.764–1.103, *p* = 0.360) by using 84 SNPs. Heterogeneity was observed by Cochran Q test (*p* < 0.001 for IVW) and two outlier SNPs were found by MR-PRESSO (Table 4). Therefore, after removing these two outliers (rs28374650 and rs77924615), re-performed MR analysis showed that serum 25-hydroxyvitamin D significantly reduced anemia risk (*OR* = 0.864, 95%*CI*: 0.757–0.986, *p* = 0.030) with the IVW method. The other four MR models all provided causal effect estimates consistent in direction with the IVW model, although the results were not statistically significant (Table 3). After removing the outliers, the MR-Egger intercept indicated no significant horizontal pleiotropy (*p* = 0.907) and no heterogeneity was observed by the Cochran Q test with a *p*-value of 0.183 for IVW (Table 4). The scatter plot of the causal association between serum 25-hydroxyvitamin D and anemia is shown in Figure S7. The leave-one-out sensitivity analysis demonstrated the robustness of the results (Figure S8). In addition, we did not find possible confounded SNPs by using LDTrait tool. A reverse MR analysis of serum 25-hydroxyvitamin D and anemia was performed and none of the five models found the presence of causal associations (*p* > 0.05), excluding the possibility of reverse causal association (Table 5).

### 3.7. Causal Relationship Between Serum Retinol Content and Anemia

IVW showed that there was no potential causal effect of serum retinol content on the risk of anemia (*OR* = 1.031, 95%*CI*: 0.914–1.164, *p* = 0.617) by using six SNPs. Risk estimates were similar for MR-Egger method, weighted median method, weighted mode method, and simple mode method (Table 3). The MR-Egger intercept indicated no significant horizontal pleiotropy (*p* = 0.557) and no heterogeneity was observed by the Cochran Q test with a *p*-value of 0.658 for IVW (Table 4). The scatter plot of the causal association between serum retinol content and anemia is shown in Figure S9. The leave-one-out sensitivity analysis demonstrated the robustness of the results (Figure S10).

### 3.8. Causal Relationship Between Erythrocyte Zinc and Anemia

Because there were only two IVs for erythrocyte zinc, only the IVW model was used to estimate the causal association between erythrocyte zinc and anemia, and horizontal pleiotropy test or leave-one-out analyses could not be performed (Table 3). IVW showed that there was no potential causal effect of erythrocyte zinc on the risk of anemia (*OR* = 1.027, 95%*CI*: 0.953–1.107, *p* = 0.491). No heterogeneity was observed by the Cochran Q test with a *p*-value of 0.947 for IVW (Table 4). The scatter plot of the causal association between erythrocyte zinc and anemia is shown in Figure S11.

## 4. Discussion

In this study, we first used nutritional health surveillance data from Hunan Province, China, to explore the potential influencing factors of anemia in school-aged children and further analyzed the associations between the four common micronutrients and anemia and then used summary data from GWAS to investigate the relationship between the four common micronutrients and the risk of anemia. The observational data indicated that daytime outdoor activity time, weekly milk consumption frequency, school location, picky eating, and serum ferritin deficiency were potential influences on anemia in school-aged children. Associations were found between serum vitamin A and serum ferritin levels and anemia. MR analyses indicated causal associations between genetically predicted serum 25-hydroxyvitamin D, serum ferritin, and serum iron levels and anemia risk, which excluded the possibility of reverse causality.

Our findings demonstrate that daytime outdoor activity durations of 30–59 min and ≥60 min were associated with 63.1% and 47.4% reduced anemia risk, respectively, compared to <30 min. This finding suggests a protective effect of moderate outdoor activity against anemia in students. However, previous studies have focused more on exercise-induced anemia caused by excessive exercise. Excessive exercise may increase the risk of anemia through several mechanisms, such as mechanical damage to erythrocytes, disturbances in iron metabolism, and inhibition of iron absorption due to inflammatory responses [49]. Unlike excessive exercise, the negative correlation between outdoor activity time and anemia risk observed in this study may reflect the positive health effects of moderate outdoor activity. The 2024–2035 master plan on building China into a leading country in education specified that primary and secondary school students in China should spend no less than 2 h per day on comprehensive physical activity. The results of this study further provide data support for this policy recommendation, confirming the importance of moderate outdoor activities on health. Students who drank milk on a weekly frequency of not daily and daily had a 38.2% and 52.9% lower risk of anemia, respectively, compared to those who barely drink milk. This result is consistent with several observational studies [10,50], indicating that milk intake significantly reduces the risk of anemia. In addition, Lien do TK et al. [51] conducted a 6-month milk intervention trial with 454 rural Vietnamese children aged 7–8 years in 2009 and showed that the prevalence of anemia was significantly reduced in the milk intervention group. Milk is an important source of high-quality protein that promotes the production and repair of red blood cells, and milk intake indirectly reduces the risk of anemia by helping to strengthen the immune system and improve overall health. Despite the positive effect of milk intake on the prevention of anemia, some studies have pointed out that milk has low iron content and low iron absorption and utilization, and excessive intake of milk may lead to inhibition of iron absorption, thereby increasing the risk of IDA [52]. Therefore, further studies are needed to verify the association between milk intake and anemia. Given that both daytime outdoor activity time and milk consumption are behavioral factors that are relatively easy to regulate and are supported by relevant policies, schools should strengthen their efforts to control these two factors. Students whose school location was rural towns/villages had a 2.133-fold increased risk of anemia compared to those in the county town, which is consistent with several studies [5,53], indicating that the prevalence of anemia among students in rural areas is generally higher than that of urban students. This may be related to insufficient dietary nutritional supply, poor nutritional health education, and unbalanced dietary structure due to the poor economy, as well as the relatively poor school conditions and the high number of boarding students due to a large proportion of their parents working outside the home, which makes it difficult for schools to satisfy the nutritional needs of the students and to provide them with nutritional guidance. Students who were picky eaters had a 1.697-fold increased risk of anemia compared to students who were not picky eaters, which is consistent with several studies [54,55], suggesting that picky eating is an important risk factor for anemia. Picky eating may lead to unbalanced nutritional intake, especially inadequate intake of key nutrients such as iron, vitamin D, and folate, which may affect erythropoiesis and function. However, the causes of picky eating may be complex and varied, including psychological factors, family environment, and dietary habits. Therefore, targeting the causes of picky eating can be more effective in improving anemia in students.

In the present study, after adjusting for covariates, students in the highest serum vitamin A quartile (Q4: ≥0.44 μg/mL) exhibited a 45.2% reduced anemia risk compared to group Q1 (<0.33 μg/mL) with a statistically significant linear trend (*P*_trend_ = 0.027), suggesting that serum vitamin A is a protective factor for anemia. This result is consistent with previous studies that elevated vitamin A levels can reduce the risk of anemia [56,57]. However, the present study did not find a significant causal effect between serum vitamin A and anemia using MR analysis. There are no MR studies on vitamin A and anemia but, in an MR study by Jingkui Zhu et al. [58], the mediating effect of vitamin A-to-oleoyl-linoleoyl-glycerol ratio was found to attenuate the effect of triacylglycerol levels on aplastic anemia. Considering the limited sample size of existing GWAS studies of serum vitamin A, with only eight available IVs, the MR findings in this study need to be further confirmed. Vitamin A is an essential micronutrient during growth and development and plays an important role in maintaining normal visual function, immune function, and epithelial cell differentiation in the body. The association between vitamin A and anemia has been widely demonstrated in population and animal studies, but the biological mechanisms of the association have not been fully elucidated. The available evidence indicates that the role of vitamin A in anemia pathogenesis is primarily associated with the regulation of erythropoiesis, modulation of iron metabolism, and enhancement of immune function to mitigate infection risk. Studies have demonstrated that vitamin A deficiency may impair the utilization of stored iron in macrophages and the liver, thereby compromising Hb synthesis [24]. Additionally, vitamin A can indirectly regulate serum iron homeostasis by modulating the expression of hepcidin mRNA. Hepcidin, a key systemic iron-regulating hormone, maintains iron demand homeostasis during Hb synthesis in erythroid precursor cells [21]. Insufficient vitamin A levels lead to reduced hepcidin expression, which, in turn, inhibits ferroportin 1 expression. This impairs cellular iron release into the bloodstream, resulting in decreased serum iron levels and subsequent anemia [23]. Vitamin A and its metabolite retinoic acid also play pivotal roles in cellular repair and immunomodulation, thereby enhancing the body’s resistance to infections [22]. For children, vitamin A is particularly critical for maintaining immune function. A robust immune system not only helps prevent autoimmune diseases but also effectively defends against exogenous pathogens, thus reducing the risk of infection-induced anemia. In conclusion, the present study identified a significant negative association between vitamin A and anemia in the student population, providing new evidence to support related research. However, the MR finding requires further validation in larger-scale GWAS.

The present study found no significant association between serum vitamin D and anemia after adjusting for covariates, which is consistent with some studies that vitamin D levels are not associated with the risk of anemia [59,60]. However, some studies have found an association between vitamin D and anemia in student populations. Bahareh Nikooyeh et al. [61] conducted a cross-sectional study involving 937 students aged 9–12 years and found that students with vitamin D deficiency had a 3.45-fold higher risk of developing anemia compared to those with normal vitamin D levels. Furthermore, in the population of US adults, serum 25-hydroxyvitamin D was found to exhibit a significant dose–response relationship with anemia and IDA and the lowest prevalence of both conditions was observed at a serum 25-hydroxyvitamin D concentration of 65.0 nmol/L [30,35]. The results of the present study may be influenced by factors such as the study population, sample size, and the methods used for sample detection. However, a causal relationship between genetically predicted serum 25-hydroxyvitamin D levels and reduced risk of anemia was found using MR analysis with no evidence of a reverse causal association. Previous MR studies have also confirmed that higher serum 25-hydroxyvitamin D levels exert a protective causal effect against multiple specific types of anemia [29,35]. The GWAS data for serum 25-hydroxyvitamin D levels used in the present study represents the most widely utilized dataset with the largest sample size to date. When combined with the latest anemia GWAS data from the Finnish database, these datasets confer high confidence to the findings of this study and provide novel evidence supporting the association between serum 25-hydroxyvitamin D and overall anemia (non-specific anemia types). Vitamin D has long been recognized for its role in regulating calcium, phosphorus, and bone metabolism. However, in recent years, growing attention has been paid to its involvement in immune function, cellular proliferation, and cardiovascular regulation, along with its strong association with the pathogenesis of various chronic diseases—including anemia [30,62]. Although population-based studies on the association between vitamin D and anemia have yielded inconsistent results, the available evidence tends to support that vitamin D deficiency contributes to the development of anemia. Nevertheless, the mechanisms underlying this association remain incompletely elucidated. Existing studies indicate that these mechanisms are closely linked to vitamin D’s roles in regulating erythropoiesis and iron metabolism. Vitamin D can directly act on erythroid precursor cells to promote the proliferation of erythroid hematopoietic stem cells, thereby participating in the regulation of erythropoiesis [19]. Additionally, it may further influence erythropoiesis by upregulating the mRNA transcription and tissue protein expression of erythropoietin receptors [18]. In terms of iron metabolism, vitamin D exerts regulatory effects primarily through modulating serum ferritin concentrations. Studies have demonstrated that vitamin D supplementation may reduce the incidence of anemia by inhibiting hepcidin mRNA expression, which, in turn, lowers serum hepcidin levels [17]. In summary, the present study provided novel evidence supporting a relationship between serum 25-hydroxyvitamin D and overall anemia using MR analysis.

In the present study, after adjusting for covariates, compared with the Q1 group, the risk of anemia in the Q3 and Q4 groups of serum ferritin levels was reduced by 39.1% and 53.0%, respectively, with a statistically significant linear trend (*P*_trend_ = 0.011). This suggests that elevated serum ferritin levels may serve as a protective factor against anemia. The RCS revealed a significant J-shaped non-linear relationship between serum ferritin and anemia risk, with a cut-off value of 36.696 µg/L. When serum ferritin levels were below 36.366 µg/L, the risk of anemia decreased significantly as ferritin levels increased. Above 36.696 µg/L, the decline in anemia risk slowed and plateaued. This suggests that serum ferritin deficiency is significantly associated with anemia but excessively high serum ferritin levels have limited impact on anemia. Since this study did not exclude the possibility of recent inflammation, elevated serum ferritin levels may also be influenced by inflammatory factors. This finding provides new evidence supporting the association between serum ferritin and anemia in the student population and is consistent with previous studies [63]. Furthermore, in the present study, MR analysis was used to investigate the causal associations between four distinct iron biomarkers and anemia. The results revealed that serum iron and serum ferritin have significant causal associations with a reduced risk of anemia, with no evidence of a reverse causal association. In previous studies, Xianjun Huang et al. [36] used MR to identify causal associations between serum iron, serum ferritin, transferrin saturation, and a reduced risk of IDA. In another study, Dipender Gill et al. [37] demonstrated that serum ferritin is causally associated with a lower risk of several anemia subtypes (aplastic anemia, IDA, and acute posthemorrhagic anemia). In contrast, the present study focused on overall anemia rather than a single subtype, which extends the causal inference of serum iron and serum ferritin to the broader condition of anemia, adding new evidence for their causal effects on overall anemia risk. As one of the most abundant trace elements in the body, iron plays an important role in Hb synthesis as well as myoglobin oxygen transport, which predisposes the body to anemia when iron absorption or intake is inadequate [64]. Serum ferritin, as a glycoprotein with strong iron storage capacity, can provide iron to the organism in a timely manner and is not affected by recent iron intake, so it can better reflect the body’s iron reserves [65]. The present study not only reconfirmed the association between serum ferritin levels and anemia but also revealed a dose–response relationship. Furthermore, MR analysis also confirmed the existence of a causal association.

The present study found no significant association between serum zinc and anemia after adjusting for covariates, which is consistent with the findings of Xiaoyu Yu et al. in a study of children aged 0–14 years [28]. However, Lisa A Houghton et al. [66] found in a study of 503 New Zealand students aged 5–15 years that low serum zinc was an independent risk factor for student anemia and mediated the effect of low selenium on Hb. Halil Ibrahim Atasoy et al. [67] identified in a case–control study that serum zinc exerted the strongest influence on Hb and served as a strong predictor of student anemia. Differences may stem from population variations and sample testing methods. Furthermore, no significant causal relationship was identified when using MR to assess the causal effect of serum zinc on anemia. No other MR studies on erythrocyte zinc and anemia exist. Given the limited sample size of existing zinc-related GWAS studies, only two IVs were available, necessitating further validation of this MR study’s findings. Zinc is a major human trace element involved in numerous coenzyme structures, playing crucial roles in growth, cognitive development, and immune function maintenance [68]. Although this study did not identify a significant association between serum zinc and anemia, existing research indicates that both zinc deficiency and excess may lead to anemia, and anemia itself may cause abnormal zinc levels in the blood [69]. The mechanisms underlying the association between zinc and anemia remain incompletely elucidated. Animal studies indicate zinc is essential for erythropoiesis and zinc deficiency disrupts red blood cell production, while zinc supplementation both in vivo and in vitro induces erythrocyte generation in rats [70,71]. Additionally, some scholars propose that zinc deficiency alone does not cause anemia. It may contribute to the onset and progression of anemia when combined with other deficiencies (such as iron, folate, or vitamin B12). However, this hypothesis requires further validation [69].

## 5. Strengths and Limitations

The present study has several strengths. First, we utilized monitoring data from a national program to comprehensively explore potential factors influencing anemia among students, providing a reference for further improving the status of student anemia. Second, we investigated the associations between four common micronutrients and anemia using both cross-sectional and MR approaches. The application of the MR design enabled us to simulate randomized controlled trials in observational studies, while avoiding the confounding effects and reverse causality.

However, the present study also has some limitations. First, the cross-sectional data only represent students in regions of Hunan Province where the NIPRCES is implemented, which may affect the representativeness of the sample. Second, the cross-sectional design is susceptible to recall bias, potentially affecting the accuracy of the results. Additionally, micronutrient monitoring in this study relied on single indicators (serum vitamin A, vitamin D, zinc, and ferritin). Although these indicators are commonly used in clinical practice and the detection method is highly sensitive, they still have some limitations and cannot comprehensively reflect the overall level of micronutrients in the body. At the same time, the possibility of inflammation in students cannot be ruled out. Since inflammation can affect iron metabolism parameters, it may lead to inaccurate serum ferritin levels. Finally, based on the cross-sectional design, this study could not directly infer causality. Although supplementary analyses were performed using the MR method, due to the lack of GWAS data from the Chinese population, the MR results could only reflect causality in the European population. Future studies should expand and validate MR analyses in Asian or other populations. This will help further clarify the value of the results across different ethnicities.

## 6. Conclusions

In summary, this study found that daytime outdoor activity time, weekly milk consumption frequency, school location, picky eating, and serum ferritin deficiency are closely associated with anemia in school-aged children. Additionally, serum vitamin A, vitamin D, serum iron, and serum ferritin levels are also linked to anemia. These findings collectively highlight the importance of lifestyle factors and specific micronutrients in influencing anemia among school-aged children, providing valuable insights for targeted prevention and intervention strategies. Future intervention trials focusing on these key factors could further validate their practical application value.

## Figures and Tables

**Table 1:** Baseline characteristics of participants by anemia status.

Characteristics	Total (n = 1725)	Anemia Status		*χ*^2^/*t*	*p* Value
		Yes (n = 155)	No (n = 1570)		
Gender				3.772	0.052
Male	874 (50.7)	67 (7.7)	807 (92.3)		
Female	851 (49.3)	88 (10.3)	763 (89.7)		
Ethnicity				2.508	0.113
Han	1301 (75.4)	125 (9.6)	1176 (90.4)		
Minority	424 (24.6)	30 (7.1)	394 (92.9)		
School segments				2.091	0.148
Primary school	952 (55.2)	77 (8.1)	875 (91.9)		
Middle school	773 (44.8)	78 (10.1)	695 (89.9)		
Age (years)				3.161	0.206
6–10	651 (37.7)	66 (10.1)	585 (89.9)		
11–13	796 (46.1)	61 (7.7)	735 (92.3)		
14–17	278 (16.1)	28 (10.1)	250 (89.9)		
Nutritional status				3.823	0.430
Normal weight	1144 (66.3)	109 (9.5)	1035 (90.5)		
Growth retardation	4 (0.2)	1 (25.0)	3 (75.0)		
Underweight	99 (5.7)	8 (8.1)	91 (91.9)		
Overweight	211 (12.2)	13 (6.2)	198 (93.8)		
Obesity	267 (15.5)	24 (9.0)	243 (91.0)		
Left behind-children				1.677	0.195
Yes	905 (7.7)	89 (9.8)	816 (90.2)		
No	820 (7.7)	66 (8.0)	754 (92.0)		
Common micronutrient levels					
Serum vitamin A (μg/mL)	0.39 ± 0.09	0.38 ± 0.10	0.39 ± 0.09	1.892	0.059
Serum vitamin D (ng/mL)	18.99 ± 5.88	18.21 ± 5.57	19.06 ± 5.90	1.720	0.086
Serum ferritin (µg/L)	68.31 ± 49.42	58.70 ± 43.78	69.26 ± 49.85	2.542	0.011
Serum zinc (µmol/L)	9.39 ± 5.63	8.98 ± 4.90	9.44 ± 5.69	0.972	0.331

**Table 2:** Associations between common micronutrient levels and the risk of anemia.

Variables	Model 1 ^a^		Model 2 ^b^		Model 3 ^c^	
	*OR* (95%*CI*)	*p*	*OR* (95%*CI*)	*p*	*OR* (95%*CI*)	*p*
Serum vitamin A (μg/mL)	0.167 (0.026–1.067)	0.059	0.158 (0.023–1.076)	0.059	0.215 (0.030–1.535)	0.125
Serum vitamin A quartile (μg/mL)						
Q1 (<0.33)	1.000 (Ref)		1.000 (Ref)		1.000 (Ref)	
Q2 (0.33–0.38)	0.712 (0.453–1.121)	0.142	0.695 (0.438–1.102)	0.122	0.718 (0.451–1.143)	0.162
Q3 (0.38–0.44)	0.732 (0.472–1.137)	0.165	0.699 (0.446–1.097)	0.120	0.746 (0.472–1.181)	0.212
Q4 (>0.44)	0.544 (0.340–0.872)	0.012	0.514 (0.316–0.836)	0.007	0.548 (0.332–0.903)	0.018
*P*_*trend*_	0.016		0.010		0.027	
Serum vitamin D (ng/mL)	0.975 (0.947–1.004)	0.086	0.981 (0.951–1.012)	0.230	0.993 (0.961–1.025)	0.655
Serum vitamin D quartile (ng/mL)						
Q1 (<14.74)	1.000 (Ref)		1.000 (Ref)		1.000 (Ref)	
Q2 (14.74–18.69)	0.847 (0.534–1.344)	0.482	0.856 (0.536–1.369)	0.517	0.938 (0.583–1.508)	0.791
Q3 (18.69–22.73)	1.096 (0.708–1.696)	0.681	1.187 (0.754–1.868)	0.460	1.348 (0.845–2.151)	0.210
Q4 (>22.73)	0.630 (0.384–1.035)	0.068	0.697 (0.413–1.176)	0.176	0.848 (0.495–1.454)	0.549
*P*_*trend*_	0.191		0.443		0.997	
Serum ferritin (µg/L)	0.993 (0.988–0.998)	0.003	0.994 (0.989–0.999)	0.012	0.994 (0.989–0.999)	0.025
Serum ferritin quartile (µg/L)						
Q1 (<38.90)	1.000 (Ref)		1.000 (Ref)		1.000 (Ref)	
Q2 (38.90–61.03)	0.604 (0.388–0.940)	0.025	0.652 (0.414–1.028)	0.066	0.638 (0.405–1.004)	0.052
Q3 (61.03–88.43)	0.606 (0.389–0.942)	0.026	0.628 (0.397–0.993)	0.047	0.609 (0.386–0.961)	0.033
Q4 (>88.43)	0.445 (0.275–0.720)	0.001	0.481 (0.293–0.791)	0.004	0.470 (0.287–0.772)	0.003
*P*_*trend*_	0.001		0.004		0.011	
Serum zinc (µmol/L)	0.850 (0.614–1.177)	0.328	0.885 (0.639–1.224)	0.459	0.923 (0.670–1.272)	0.624
Serum zinc quartile (µmol/L)						
Q1 (<6.50)	1.000 (Ref)		1.000 (Ref)		1.000 (Ref)	
Q2 (6.50–8.20)	0.997 (0.644–1.544)	0.991	1.074 (0.688–1.677)	0.753	1.098 (0.701–1.721)	0.682
Q3 (8.20–10.20)	0.647 (0.401–1.043)	0.074	0.713 (0.438–1.159)	0.172	0.787 (0.481–1.288)	0.340
Q4 (>10.20)	0.740 (0.464–1.181)	0.207	0.836 (0.515–1.356)	0.467	0.916 (0.561–1.495)	0.726
*P*_*trend*_	0.076		0.215		0.447	

^a^ Model 1 was unadjusted; ^b^ Model 2 was adjusted for sex, ethnicity, school segments, age, nutritional status, and left-behind children. ^c^ Model 3 further adjusts other common micronutrients (serum vitamin A, serum vitamin D, serum ferritin, and serum zinc) except for the grouping variables based on Model 2. Abbreviations: *OR*, odds ratio; *CI*, confidence interval.

**Table 3:** Mendelian randomization analysis of anemia and common micronutrients.

Phenotype	Whether to Remove Outliers	IVs	*F* Value	MR Method	*β*	Se	*p* Value	*OR* (95%*CI*)
Serum ferritin	No	55	76.487	MR Egger	−0.458	0.116	<0.001	0.633 (0.504–0.794)
				Weighted median	−0.448	0.084	<0.001	0.639 (0.542–0.753)
				IVW	−0.442	0.062	<0.001	0.643 (0.570–0.726)
				Simple mode	−0.471	0.185	0.014	0.625 (0.434–0.898)
				Weighted mode	−0.471	0.102	<0.001	0.625 (0.511–0.763)
Serum ferritin	Yes	54	76.841	MR Egger	−0.439	0.104	<0.001	0.645 (0.526–0.790)
				Weighted median	−0.447	0.084	<0.001	0.640 (0.543–0.754)
				IVW	−0.422	0.055	<0.001	0.656 (0.588–0.731)
				Simple mode	−0.464	0.178	0.012	0.629 (0.443–0.892)
				Weighted mode	−0.471	0.106	0.000	0.624 (0.507–0.768)
Serum iron	No	21	129.068	MR Egger	−0.118	0.145	0.427	0.889 (0.669–1.181)
				Weighted median	−0.210	0.093	0.024	0.811 (0.676–0.973)
				IVW	−0.197	0.090	0.029	0.821 (0.688–0.980)
				Simple mode	−0.236	0.195	0.240	0.790 (0.539–1.157)
				Weighted mode	−0.201	0.097	0.052	0.818 (0.675–0.990)
Serum iron	Yes	19	130.911	MR Egger	−0.146	0.118	0.234	0.864 (0.686–1.090)
				Weighted median	−0.235	0.093	0.012	0.790 (0.658–0.949)
				IVW	−0.231	0.078	0.003	0.793 (0.681–0.925)
				Simple mode	−0.242	0.178	0.192	0.785 (0.554–1.114)
				Weighted mode	−0.228	0.091	0.022	0.796 (0.666–0.952)
Total iron-binding capacity	No	23	63.239	MR Egger	0.388	0.347	0.276	1.474 (0.747–2.907)
				Weighted median	0.153	0.112	0.169	1.166 (0.937–1.451)
				IVW	0.188	0.116	0.106	1.207 (0.961–1.515)
				Simple mode	0.097	0.209	0.647	1.102 (0.732–1.658)
				Weighted mode	0.182	0.204	0.381	1.200 (0.804–1.790)
Total iron-binding capacity	Yes	21	64.098	MR Egger	−0.203	0.288	0.490	0.816 (0.464–1.436)
				Weighted median	0.152	0.111	0.171	1.164 (0.937–1.446)
				IVW	0.069	0.091	0.447	1.071 (0.897–1.280)
				Simple mode	0.125	0.218	0.574	1.133 (0.739–1.736)
				Weighted mode	0.219	0.225	0.344	1.244 (0.800–1.935)
Transferrin saturation	No	18	161.776	MR Egger	−0.102	0.124	0.423	0.903 (0.708–1.152)
				Weighted median	−0.126	0.070	0.072	0.882 (0.769–1.011)
				IVW	−0.115	0.071	0.103	0.891 (0.776–1.024)
				Simple mode	−0.046	0.159	0.774	0.955 (0.699–1.303)
				Weighted mode	−0.149	0.067	0.041	0.861 (0.755–0.983)
Transferrin saturation	Yes	16	90.621	MR Egger	0.278	0.163	0.110	1.321 (0.960–1.816)
				Weighted median	0.111	0.092	0.225	1.118 (0.934–1.338)
				IVW	0.008	0.081	0.920	1.008 (0.860–1.181)
				Simple mode	0.018	0.175	0.918	1.019 (0.723–1.434)
				Weighted mode	0.133	0.094	0.179	1.142 (0.950–1.373)
Serum 25-hydroxyvitamin D	No	84	94.495	MR Egger	−0.217	0.178	0.227	0.805 (0.567–1.142)
				Weighted median	−0.156	0.116	0.178	0.855 (0.681–1.074)
				IVW	−0.086	0.094	0.360	0.918 (0.764–1.103)
				Simple mode	−0.078	0.204	0.705	0.925 (0.620–1.381)
				Weighted mode	−0.055	0.118	0.642	0.946 (0.751–1.193)
Serum 25-hydroxyvitamin D	Yes	82	95.867	MR Egger	−0.133	0.129	0.304	0.875 (0.680–1.127)
				Weighted median	−0.157	0.110	0.155	0.855 (0.689–1.061)
				IVW	−0.146	0.068	0.030	0.864 (0.757–0.986)
				Simple mode	−0.067	0.213	0.752	0.935 (0.616–1.419)
				Weighted mode	−0.055	0.114	0.633	0.947 (0.757–1.184)
Serum retinol content	No	6	45.982	MR Egger	−0.117	0.239	0.651	0.890 (0.558–1.421)
				Weighted median	0.034	0.078	0.658	1.035 (0.889–1.205)
				IVW	0.031	0.062	0.617	1.031 (0.914–1.164)
				Simple mode	0.135	0.109	0.271	1.144 (0.924–1.417)
				Weighted mode	0.038	0.102	0.727	1.038 (0.850–1.268)
Erythrocyte Zinc	No	2	61.254	MR Egger	NA	NA	NA	NA
				Weighted median	NA	NA	NA	NA
				IVW	0.026	0.038	0.491	1.027 (0.953–1.107)
				Simple mode	NA	NA	NA	NA
				Weighted mode	NA	NA	NA	NA

Abbreviations: *OR*, odds ratio; *CI*, confidence interval; IVs, instrumental variables; IVW, inverse variance weighted; MR, Mendelian randomization. “NA” indicates that the corresponding value cannot be calculated due to an insufficient number of instrumental variables.

**Table 4:** Sensitivity analysis of the Mendelian randomization analysis results of anemia and common micronutrients.

Phenotype	Whether to Remove Outliers	Method	Heterogeneity			Pleiotropy		
			*Q* Value	*df*	*p* Value	egg_intercept	Se	*p* Value
Serum ferritin	No	MR Egger	78.787	53	0.012	0.001	0.004	0.869
		IVW	78.828	54	0.015	-	-	-
Serum ferritin	Yes	MR Egger	61.460	52	0.173	0.001	0.004	0.848
		IVW	61.504	53	0.198	-	-	-
Serum iron	No	MR Egger	42.052	19	0.002	−0.006	0.008	0.490
		IVW	43.149	20	0.002	-	-	-
Serum iron	Yes	MR Egger	24.642	17	0.103	−0.007	0.007	0.346
		IVW	26.003	18	0.100	-	-	-
Total iron-binding capacity	No	MR Egger	57.451	21	0.000	−0.009	0.015	0.546
		IVW	58.480	22	0.000	-	-	-
Total iron-binding capacity	Yes	MR Egger	27.886	19	0.086	0.012	0.012	0.333
		IVW	29.337	20	0.081	-	-	-
Transferrin saturation	No	MR Egger	38.986	16	0.001	−0.001	0.009	0.900
		IVW	39.025	17	0.002	-	-	-
Transferrin saturation	Yes	MR Egger	18.501	14	0.185	−0.016	0.009	0.083
		IVW	23.120	15	0.082	-	-	-
Serum 25-hydroxyvitamin D	No	MR Egger	182.453	82	0.000	0.004	0.004	0.389
		IVW	184.121	83	0.000	-	-	-
Serum 25-hydroxyvitamin D	Yes	MR Egger	92.339	80	0.163	−3.58 × 10^−4^	0.003	0.907
		IVW	92.355	81	0.183	-	-	-
Serum retinol content	No	MR Egger	2.862	4	0.581	0.014	0.022	0.557
		IVW	3.272	5	0.658	-	-	-
Erythrocyte Zinc	No	MR Egger	NA	NA	NA	NA	NA	NA
		IVW	0.004	1	0.947	NA	NA	NA

Abbreviations: IVW, inverse variance weighted; MR, Mendelian randomization. “NA” indicates that the corresponding value cannot be calculated due to an insufficient number of instrumental variables.

**Table 5:** Reverse Mendelian randomization analysis of anemia and serum ferritin, serum iron, and serum 25-hydroxyvitamin D.

Exposure Phenotype	Outcome Phenotype	IVs	*F* Value	MR Method	*β*	Se	*p* Value	*OR* (95%*CI*)
Anemia	Serum ferritin	5	51.756	MR Egger	−0.041	0.055	0.514	0.960 (0.862–1.070)
				Weighted median	−0.023	0.025	0.355	0.977 (0.931–1.026)
				IVW	−0.012	0.021	0.563	0.988 (0.948–1.029)
				Simple mode	−0.032	0.036	0.419	0.968 (0.902–1.039)
				Weighted mode	−0.025	0.029	0.436	0.975 (0.921–1.032)
Anemia	Serum iron	6	60.016	MR Egger	0.018	0.056	0.765	1.018 (0.913–1.136)
				Weighted median	−0.009	0.027	0.723	0.991 (0.940–1.044)
				IVW	−0.032	0.020	0.106	0.969 (0.932–1.007)
				Simple mode	−0.010	0.042	0.820	0.990 (0.912–1.074)
				Weighted mode	−0.006	0.040	0.883	0.994 (0.919–1.075)
Anemia	Serum 25-hydroxyvitamin D	5	51.796	MR Egger	0.062	0.048	0.291	1.064 (0.968–1.170)
				Weighted median	0.018	0.017	0.301	1.018 (0.984–1.052)
				IVW	0.013	0.020	0.526	1.013 (0.974–1.053)
				Simple mode	0.029	0.024	0.303	1.029 (0.981–1.079)
				Weighted mode	0.031	0.019	0.173	1.031 (0.994–1.070)

Abbreviations: *OR*, odds ratio; *CI*, confidence interval; IVs, instrumental variables; IVW, inverse variance weighted; MR, Mendelian randomization.

## Data Availability

The original contributions presented in this study are included in the article/Supplementary Materials. Further inquiries can be directed to the corresponding author.

## Supplementary Materials

The following supporting information can be downloaded at: https://www.mdpi.com/article/10.3390/nu17203220/s1, Figure S1: The study design diagram of Mendelian randomization; Figure S2: Flowchart of two-sample Mendelian randomization study analysis; Figure S3: Scatter plot of the causal association between serum iron status indicators and anemia before removing the outliers; Figure S4: Scatter plot of the causal association between serum iron status indicators and anemia after removing the outliers; Figure S5: Leave-one-out analysis for the causal association between serum iron status indicators and anemia before removing the outliers; Figure S6: Leave-one-out analysis for the causal association between serum iron status indicators and anemia after removing the outliers; Figure S7: Scatter plot of the causal association between serum 25-hydroxyvitamin D and anemia; Figure S8: Leave-one-out analysis for the causal association between serum 25-hydroxyvitamin D and anemia; Figure S9: Scatter plot of the causal association between serum retinol content and anemia; Figure S10: Leave-one-out analysis for the causal association between serum retinol content and anemia; Figure S11: Scatter plot of the causal association between erythrocyte zinc and anemia; Table S1: Summary of basic information on GWAS data; Table S2: Prevalence of anemia by different micronutrient levels and 95% confidence interval; Table S3: Univariate logistic regression model for risk factors associated with anemia; Table S4: The results of multi-collinearity diagnosis; Table S5: Subgroup analysis of the association between serum vitamin A and anemia; Table S6: Subgroup analysis of the association between serum ferritin and anemia.

## Abbreviations

BMI: body mass index; CI: confidence interval; GWAS: genome-wide association study; Hb: hemoglobin; IDA: iron deficiency anemia; IVs: instrumental variables; IVW: inverse variance weighted; MAF: minor allele frequencies; MR: Mendelian randomization; MR-PRESSO: MR-Pleiotropy Residual Sum and Outlier methods; NIPRCES: Nutrition Improvement Programme for Rural Compulsory Education Students; OR: odds ratio; RCS: restricted cubic splines; SNP: single-nucleotide polymorphism; VIF: variance inflation factor; WHO: World Health Organization; YLDs: years lost due to disability.
