# Supplementary material for: Vitamin A, Vitamin D, Iron, and Zinc in Relation to Anemia Risk: Observational Evidence and Mendelian Randomization
Source: Nutrients. 2025 Oct 14;17(20):3220. doi: 10.3390/nu17203220 (PMC12567539; doi:10.3390/nu17203220)
Supplement: Supplementary file 1 [file nutrients-17-03220-s001.zip › nutrients-3873225-supplementary.pdf]

Attachment information

Table S1 Summary of basic information on GWAS data

Phenotype	Year of publication	Population	Sample size	Number of SNPs	Web source
Anemia	2024	European	138,342	21278080	https://r11.finngen.fi/
Serum 25-Hydroxyvitamin D	2020	European	417,580	8806780	https://cnsgenomics.com/content/data
Serum ferritin	2021	European	246,139	21642116	https://www.decode.com/summarydata/
Serum iron	2021	European	163,511	19689928	https://www.decode.com/summarydata/
Total iron binding capacity	2021	European	135,430	19293087	https://www.decode.com/summarydata/
Transferrin saturation	2021	European	131,471	19330058	https://www.decode.com/summarydata/
Erythrocyte Zinc	2013	European	2603	2540325	https://gwas.mrcieu.ac.uk/datasets/ieu-a-1079/
Serum retinol content	2024	European	17,268	8173975	https://doi.org/10.5281/zenodo.7905523

Abbreviations: SNP, Single nucleotide polymorphism.

Table S2 Prevalence of anemia by different micronutrient levels and 95%confidence interval

Variables	Anemia % (95%*CI*)
Serum vitamin A (μg/mL)	8.99 (7.63-10.34)
Serum vitamin A quartile (μg/mL)	
Q1 (<0.33)	11.72 (8.69-14.76)
Q2 (0.33-0.38)	8.64 (5.89-11.39)
Q3 (0.38-0.44)	8.86 (6.20-11.53)
Q4 (>0.44)	6.74 (4.40-9.08)
Serum vitamin D (ng/ml)	8.99 (7.63-10.34)
Serum vitamin D quartile (ng/ml)	
Q1 (<14.74)	9.98 (7.14-12.82)
Q2 (14.74-18.69)	8.58 (5.93-11.24)
Q3 (18.69-22.73)	10.83 (7.89-13.76)
Q4 (>22.73)	6.53 (4.18-8.87)
Serum ferritin (μg/L)	8.99 (7.63-10.34)
Serum ferritin quartile (μg/L)	
Q1 (<38.90)	13.05 (9.85-16.25)
Q2 (38.90-61.03)	8.31 (5.70-10.92)
Q3 (61.03-88.43)	8.33 (5.72-10.95)
Q4 (>88.43)	6.26 (3.97-8.56)
Serum zinc (μmol/L)	8.99 (7.63-10.34)
Serum zinc quartile (μmol/L)	
Q1 (<6.50)	10.49 (7.58-13.40)
Q2 (6.50-8.20)	10.47 (7.56-13.37)
Q3 (8.20-10.20)	7.05 (4.64-9.45)
Q4 (>10.20)	7.98 (5.40-10.57)

Abbreviations: *CI*, confidence interval.

Table S3 Univariate logistic regression model for risk factors associated with anemia

Characteristics	Number (n)	Anemia [n (%)]	*β*	*SE*	Waldχ2	*P* value	*OR* (95%*CI*)
Basic information							
Sex							
Male	874	67 (7.7)					
Female	851	88 (10.3)	0.329	0.170	3.747	0.053	1.389 (0.996-1.938)
Ethnicity							
Han	1301	125 (9.6)					
Minority	424	30 (7.1)	-0.334	0.211	2.488	0.115	0.716 (0.473-1.084)
School segments							
Primary school	952	77 (8.1)					
Middle school	773	78 (10.1)	0.243	0.168	2.084	0.149	1.275 (0.917-1.774)
Age (years)							
6-10	651	66(10.1)					
11-13	796	61(7.7)	-0.307	0.186	2.723	0.099	0.736 (0.511-1.059)
14-17	278	28(10.1)	-0.007	0.238	0.001	0.976	0.993 (0.623-1.582)
Nutritional status							
Normal weight	1144	109 (9.5)					
Growth retardation	4	1 (25.0)	1.152	1.159	0.988	0.320	3.165 (0.326-30.691)
Under weight	99	8 (8.1)	-0.181	0.382	0.223	0.637	0.835 (0.395-1.766)
Overweight	211	13 (6.2)	-0.473	0.304	2.424	0.120	0.623 (0.344-1.130)
Obesity	267	24 (9.0)	-0.064	0.236	0.074	0.786	0.938 (0.590-1.491)
Left behind-children							
Yes	905	89 (9.8)					
No	820	66 (8.0)	-0.220	0.170	1.672	0.196	0.803 (0.575-1.120)

Exercise and Dietary Behaviors							
Daytime outdoor activity time (minutes)							
<30	189	30 (15.9)					
30-59	660	44 (6.7)	-0.971	0.253	14.749	**<0.001**	0.379 (0.231-0.622)
≥60	876	81 (9.2)	-0.616	0.231	7.133	**0.008**	0.540 (0.344-0.849)
Daily moderate-to-vigorous physical activity Time							
<30	649	62 (9.6)					
30-59	753	65 (8.6)	-0.112	0.186	0.359	0.549	0.894 (0.621-1.288)
≥60	323	28 (8.7)	-0.107	0.239	0.201	0.654	0.899 (0.563-1.434)
Daily meal frequency (times)							
<3	409	37 (9.0)					
≥3	1316	118 (9.0)	-0.010	0.198	0.002	0.961	0.990 (0.672-1.459)
Picky eating							
NO	1062	78 (7.3)					
Yes	663	77 (11.6)	0.505	0.169	8.953	**0.003**	1.658 (1.190-2.308)
Weekly meat consumption frequency							
Barely eat	39	5 (12.8)					
Not daily	912	87 (9.5)	-0.333	0.492	0.457	0.499	0.717 (0.273-1.881)
Daily	774	63 (8.1)	-0.507	0.497	1.040	0.308	0.603 (0.228-1.595)
Weekly eggs consumption frequency							
Barely eat	164	14 (8.5)					
Not daily	1279	124 (9.7)	0.140	0.295	0.225	0.635	1.150 (0.645-2.051)
Daily	282	17 (6.0)	-0.375	0.375	0.999	0.318	0.687 (0.330-1.434)
Weekly milk consumption frequency							
Barely drink	234	32 (13.7)					
Not daily	955	8.9 (9.3)	-0.433	0.220	3.853	**0.050**	0.649 (0.421-0.999)

Daily	536	34 (6.3)	-0.850	0.260	10.680	**0.001**	0.428 (0.257-0.712)
Weekly legumes consumption frequency							
Barely eat	258	21 (8.1)					
Not daily	1323	128 (9.7)	0.190	0.246	0.595	0.441	1.209 (0.746-1.958)
Daily	144	6 (4.2)	-0.712	0.475	2.245	0.134	0.491 (0.193-1.245)
Daily variety of fresh vegetable intake							
Barely eat	75	9 (12.0)					
<3	1336	119 (8.9)	-0.333	0.368	0.816	0.366	0.717 (0.349-1.475)
≥3	314	27 (8.6)	-0.371	0.408	0.826	0.363	0.690 (0.310-1.536)
Weekly fruits consumption frequency							
Barely eat	83	12 (14.5)					
Not daily	1199	109 (9.1)	-0.525	0.328	2.562	0.109	0.592 (0.311-1.125)
Daily	443	34 (7.7)	-0.710	0.360	3.895	**0.048**	0.492 (0.243-0.995)
Weekly snack consumption frequency							
Barely eat	183	18 (9.8)					
Not daily	1200	114 (9.5)	-0.038	0.267	0.021	0.885	0.962 (0.570-1.624)
Daily	342	23 (6.7)	-0.414	0.329	1.585	0.208	0.661 (0.347-1.259)
Weekly beverage consumption frequency							
Barely eat	780	68 (8.7)					
Not daily	851	82 (9.6)	0.110	0.172	0.410	0.522	1.116 (0.797-1.564)
Daily	94	5 (5.3)	-0.531	0.477	1.238	0.266	0.588 (0.231-1.498)
Weekly fried food consumption frequency							
Barely eat	812	84 (10.3)					
Not daily	834	68 (8.2)	-0.262	0.171	2.347	0.126	0.769 (0.550-1.076)
Daily	79	3 (3.8)	-1.073	0.600	3.198	0.074	0.342 (0.106-1.108)
Basic School Information							

Terrain							
Mountainous	1113	97 (8.7)					
Hilly	612	58 (9.5)	0.092	0.174	0.280	0.597	1.097 (0.779-1.543)
School location							
County town	954	59 (6.2)					
Rural towns/villages	771	96 (12.5)	0.769	0.173	19.731	**<0.001**	2.157 (1.537-3.029)
Daily meal frequency provided by the school (times)							
≤1	356	31 (8.7)					
2	509	42 (8.3)	-0.059	0.248	0.056	0.812	0.943 (0.580-1.532)
≥3	860	82 (9.5)	0.100	0.221	0.204	0.516	1.105 (0.717-1.704)
Does the school maintain student health records?							
Yes	1197	99 (8.3)					
No	528	56 (10.6)	0.274	0.176	2.432	0.119	1.316 (0.932-1.858)
Does the school have a cafeteria?							
Yes	1675	154 (9.2)					
No	50	1 (2.0)	-1.602	1.014	2.496	0.114	0.202(0.028-1.470)
Health education							
Yes	1310	116 (8.9)					
No	415	39 (9.4)	0.065	0.194	0.113	0.736	1.068 (0.729-1.563)
Common micronutrient levels							
Serum vitamin A							
Normal	1470	122 (8.3)					
Deficiency	255	33 (12.9)	0.496	0.209	5.628	**0.018**	1.642 (1.090-2.475)
Serum vitamin D							
Normal	712	59 (8.3)					
Insufficient	806	73 (9.1)	0.097	0.183	0.283	0.595	1.021 (0.743-1.402)

Deficiency	207	23 (11.1)	0.325	0.260	1.563	0.211	1.383 (0.832-2.301)
Serum ferritin							
Normal	1659	135 (8.1)					
Deficiency	66	20 (30.3)	1.591	0.282	31.716	**<0.001**	4.908 (2.821-8.539)
Serum zinc							
Normal	1554	135 (8.7)					
Deficiency	171	20 (11.7)	0.331	0.254	1.691	0.193	1.392 (0.846-2.292)

Abbreviations: *OR*, odds ratio; *CI*, confidence interval.

Table S4 The results of multi-collinearity diagnosis

Characteristics	Tolerance	*VIF*
Daytime outdoor activity time	0.977	1.023
Weekly milk consumption frequency	0.970	1.031
Weekly fruits consumption frequency	0.970	1.031
School location	0.975	1.026
Picky eating	0.998	1.002
Serum vitamin A levels	0.979	1.021
Serum ferritin levels	0.998	1.002

Abbreviations: *VIF*, Variance inflation factor.

Table S5 Subgroup analysis of the association between serum vitamin A and anemia

Characteristics	Serum vitamin A quartile (μg/mL)				*P* value
	Q1 (<0.33)	Q2 (0.33-0.38)	Q3 (0.38-0.44)	Q4 (>0.44)	
Age (years)					
6-10	1.00 (Ref)	**0.444 (0.223-0.885)**	**0.386 (0.183-0.811)**	**0.274 (0.113-0.663)**	**0.005**
11-13	1.00 (Ref)	1.184 (0.514-2.728)	1.598 (0.722-3.537)	1.066 (0.452-2.511)	0.601
14-17	1.00 (Ref)	1.333 (0.357-4.973)	1.166 (0.311-4.370)	0.944 (0.257-3.470)	0.942
Gender					
Male	1.00 (Ref)	**0.445 (0.217-0.913)**	0.571 (0.293-1.114)	**0.315 (0.136-0.731)**	**0.022**
Female	1.00 (Ref)	1.085 (0.560-2.101)	0.948 (0.482-1.862)	0.757 (0.381-1.507)	0.738
Nutritional status					
Normal weight	1.00 (Ref)	0.696 (0.404-1.199)	0.618 (0.351-1.088)	**0.450 (0.245-0.826)**	**0.070**
Growth retardation	1.00 (Ref)	-	-	-	-
Under weight	1.00 (Ref)	1.541 (0.066-35.801)	3.570 (0.070-181.731)	6.051 (0.143-256.906)	0.783
Overweight	1.00 (Ref)	0.719 (0.045-11.555)	5.390 (0.659-44.096)	4.157 (0.525-32.941)	0.248
Obesity	1.00 (Ref)	0.544 (0.139-2.123)	1.073 (0.320-3.595)	0.350 (0.081-1.520)	0.335
School segments					
Primary school	1.00 (Ref)	**0.501 (0.267-0.941)**	**0.487 (0.253-0.939)**	**0.291 (0.129-0.657)**	**0.008**
Middle school	1.00 (Ref)	1.164 (0.543-2.496)	1.261 (0.602-2.642)	0.964 (0.450-2.066)	0.836
Ethnicity					
Han	1.00 (Ref)	0.779 (0.459-1.321)	0.803 (0.479-1.346)	0.643 (0.37-1.118)	0.474
Minority	1.00 (Ref)	0.857 (0.287-2.558)	1.060 (0.344-3.268)	0.274 (0.067-1.124)	0.304
Left behind-children					
Yes	1.00 (Ref)	**0.514 (0.275-0.961)**	0.598 (0.327-1.091)	**0.302 (0.151-0.605)**	**0.007**
No	1.00 (Ref)	1.141 (0.547-2.379)	0.994 (0.471-2.095)	1.128 (0.524-2.430)	0.972
Serum vitamin D quartile (ng/ml)					

Q1 (<14.74)	1.00 (Ref)	0.511 (0.204-1.277)	0.995 (0.426-2.324)	0.563 (0.200-1.588)	0.366
Q2 (14.74-18.69)	1.00 (Ref)	0.647 (0.233-1.796)	0.823 (0.331-2.045)	0.328 (0.103-1.040)	0.277
Q3 (18.69-22.73)	1.00 (Ref)	1.235 (0.502-3.038)	0.654 (0.240-1.782)	0.766 (0.294-1.997)	0.535
Q4 (>22.73)	1.00 (Ref)	0.581 (0.183-1.848)	0.661 (0.217-2.020)	0.539 (0.165-1.759)	0.723
Serum ferritin quartile (μg/L)					
Q1 (<38.90)	1.00 (Ref)	0.751 (0.343-1.643)	0.551 (0.235-1.291)	**0.407 (0.170-0.979)**	0.221
Q2 (38.90-61.03)	1.00 (Ref)	0.516 (0.169-1.583)	1.335 (0.534-3.339)	0.425 (0.131-1.378)	0.118
Q3 (61.03-88.43)	1.00 (Ref)	0.650 (0.246-1.720)	0.625 (0.235-1.665)	0.567 (0.200-1.606)	0.682
Q4 (>88.43)	1.00 (Ref)	1.114 (0.359-3.452)	0.451 (0.122-1.671)	0.898 (0.281-2.868)	0.579
Serum zinc quartile (μg/dL)					
Q1 (<0.65)	1.00 (Ref)	0.667 (0.280-1.593)	0.822 (0.332-2.038)	0.491 (0.180-1.345)	0.539
Q2 (0.65-0.82)	1.00 (Ref)	1.081 (0.474-2.464)	0.936 (0.399-2.198)	**0.234 (0.063-0.860)**	0.135
Q3 (0.82-1.02)	1.00 (Ref)	0.392 (0.110-1.398)	0.796 (0.292-2.168)	0.514 (0.172-1.542)	0.429
Q4 (>1.02)	1.00 (Ref)	0.485 (0.160-1.471)	**0.303 (0.095-0.966)**	0.585 (0.206-1.665)	0.232

In the multivariate models, confounding factors such as gender, ethnicity, school segments, age, nutritional status, and left behind-children and common micronutrient quartiles (serum vitamin D, serum ferritin and serum zinc) were included unless the variable was used as a subgroup variable. Abbreviations: *OR*, odds ratio; *CI*, confidence interval.

Table S6 Subgroup analysis of the association between serum ferritin and anemia

Characteristics	Serum ferritin quartile (μg/L)				*P* value
	Q1 (<38.31)	Q2 (38.31-60.83)	Q3 (60.83-87.64)	Q4 (>87.64)	
Age (years)					
6-10	1.00 (Ref)	1.037 (0.463-2.323)	1.075 (0.479-2.409)	0.869 (0.367-2.056)	0.950
11-13	1.00 (Ref)	0.733 (0.373-1.442)	0.405 (0.178-0.920)	0.466 (0.205-1.062)	0.102
14-17	1.00 (Ref)	0.141 (0.017-1.140)	0.807 (0.285-2.282)	0.288 (0.074-1.127)	0.118
Gender					
Male	1.00 (Ref)	1.073 (0.532-2.165)	0.929 (0.444-1.942)	0.580 (0.254-1.322)	0.462
Female	1.00 (Ref)	**0.471 (0.246-0.903)**	0.568 (0.302-1.070)	**0.478 (0.243-0.938)**	0.058
Nutritional status					
Normal weight	1.00 (Ref)	0.809 (0.470-1.394)	0.785 (0.453-1.361)	**0.468 (0.247-0.887)**	0.141
Growth retardation	1.00 (Ref)	-	-	-	-
Under weight	1.00 (Ref)	-	-	-	-
Overweight	1.00 (Ref)	0.175 (0.026-1.157)	0.222 (0.019-2.620)	0.226 (0.029-1.732)	0.238
Obesity	1.00 (Ref)	0.465 (0.139-1.549)	0.529 (0.168-1.666)	**0.164 (0.032-0.858)**	0.164
School segments					
Primary school	1.00 (Ref)	1.099 (0.541-2.231)	0.937 (0.446-1.969)	0.931 (0.431-2.008)	0.958
Middle school	1.00 (Ref)	0.530 (0.269-1.047)	0.624 (0.328-1.186)	**0.344 (0.158-0.748)**	**0.031**
Ethnicity					
Han	1.00 (Ref)	0.826 (0.502-1.360)	0.764 (0.454-1.286)	0.573 (0.320-1.025)	0.307
Minority	1.00 (Ref)	**0.189 (0.046-0.772)**	0.520 (0.169-1.605)	0.394 (0.124-1.245)	0.115
Left behind-children					
Yes	1.00 (Ref)	0.762 (0.428-1.356)	0.635 (0.338-1.194)	**0.400 (0.189-0.848)**	0.105
No	1.00 (Ref)	0.521 (0.236-1.152)	0.744 (0.365-1.518)	0.617 (0.298-1.279)	0.382
Serum vitamin D quartile (ng/ml)					

Q1 (<14.74)	1.00 (Ref)	**0.337 (0.127-0.895)**	0.532 (0.225-1.256)	0.545 (0.201-1.480)	0.128
Q2 (14.74-18.69)	1.00 (Ref)	0.432 (0.169-1.103)	0.429 (0.154-1.191)	0.353 (0.123-1.014)	0.144
Q3 (18.69-22.73)	1.00 (Ref)	0.881 (0.381-2.040)	0.938 (0.386-2.281)	0.573 (0.215-1.525)	0.705
Q4 (>22.73)	1.00 (Ref)	1.814 (0.470-6.992)	1.756 (0.455-6.779)	1.078 (0.273-4.264)	0.692
Serum vitamin A quartile (μg/mL)					
Q1 (<0.33)	1.00 (Ref)	**0.396 (0.164-0.956)**	0.510 (0.214-1.216)	**0.334 (0.131-0.847)**	0.077
Q2 (0.33-0.38)	1.00 (Ref)	0.410 (0.139-1.208)	0.492 (0.180-1.342)	0.467 (0.160-1.366)	0.305
Q3 (0.38-0.44)	1.00 (Ref)	1.630 (0.670-3.970)	0.789 (0.297-2.096)	0.411 (0.119-1.417)	0.106
Q4 (>0.44)	1.00 (Ref)	0.469 (0.144-1.533)	0.831 (0.271-2.545)	0.754 (0.249-2.285)	0.654
Serum zinc quartile (μg/dL)					
Q1 (<0.65)	1.00 (Ref)	0.510 (0.216-1.205)	0.689 (0.300-1.582)	**0.119 (0.026-0.544)**	**0.036**
Q2 (0.65-0.82)	1.00 (Ref)	0.992 (0.434-2.266)	0.423 (0.155-1.152)	0.666 (0.254-1.745)	0.306
Q3 (0.82-1.02)	1.00 (Ref)	1.032 (0.347-3.072)	0.937 (0.308-2.848)	0.882 (0.283-2.749)	0.993
Q4 (>1.02)	1.00 (Ref)	0.461 (0.140-1.511)	1.094 (0.398-3.008)	0.810 (0.289-2.269)	0.497

In the multivariate models, confounding factors such as gender, ethnicity, school segments, age, nutritional status, and left behind-children and common micronutrient quartiles (serum vitamin D, Serum vitamin A and serum zinc) were included unless the variable was used as a subgroup variable. Abbreviations: *OR*, odds ratio; *CI*, confidence interval.

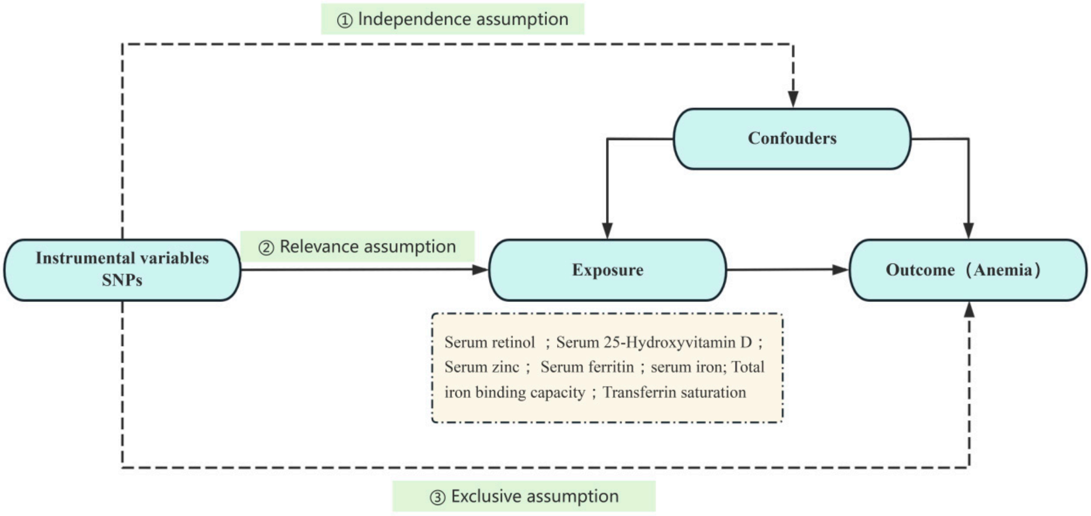

Fig. S1 The study design diagram of Mendelian randomization.Abbreviations: SNP, Single nucleotide polymorphism.

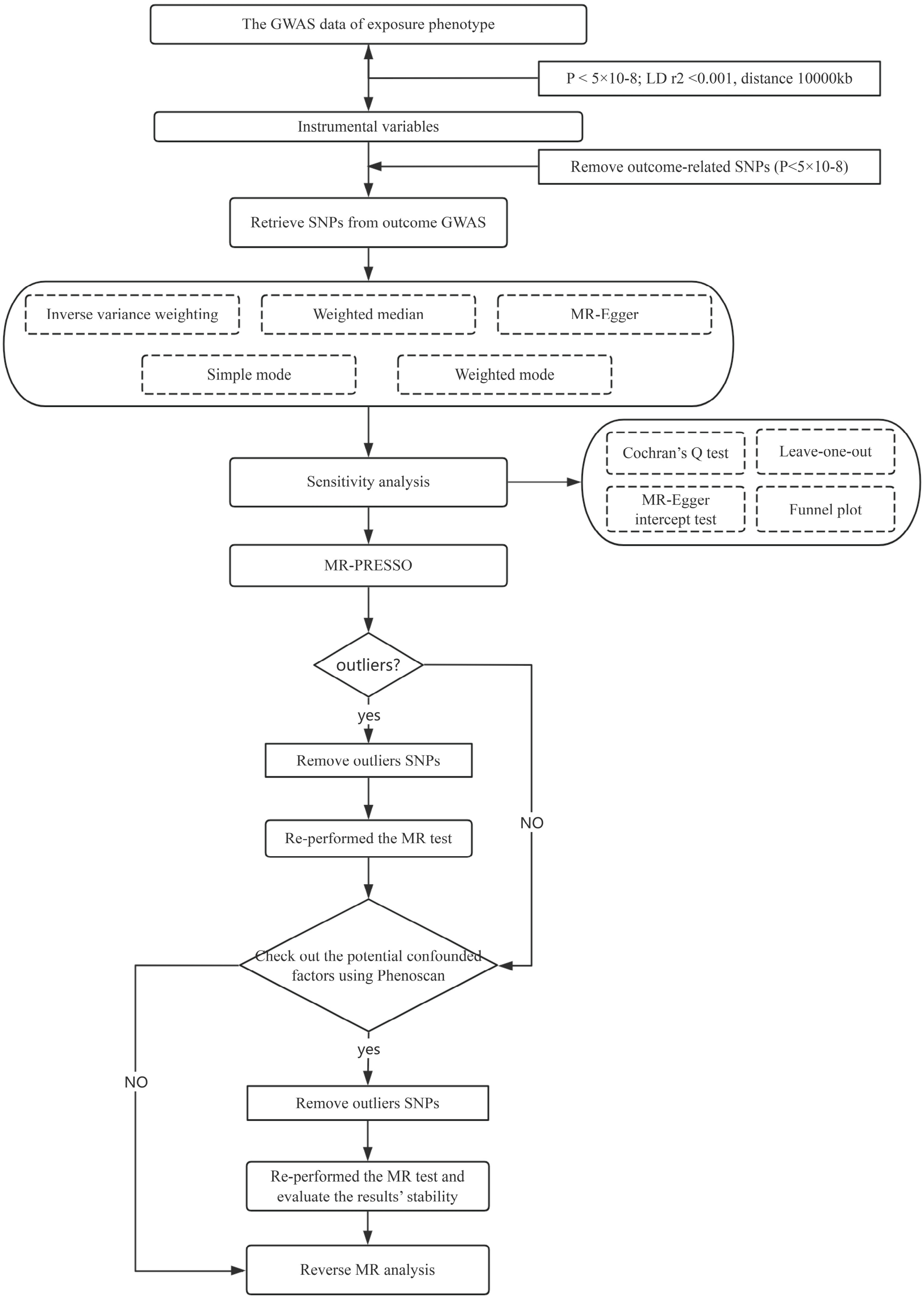

Fig. S2 Flowchart of two-sample Mendelian randomization study analysis. Abbreviations: SNP, Single nucleotide polymorphism; GWAS, Genome-wide association study; MR-PRESSO, MR-Pleiotropy Residual Sum and Outlier methods; MR, Mendelian Randomization.

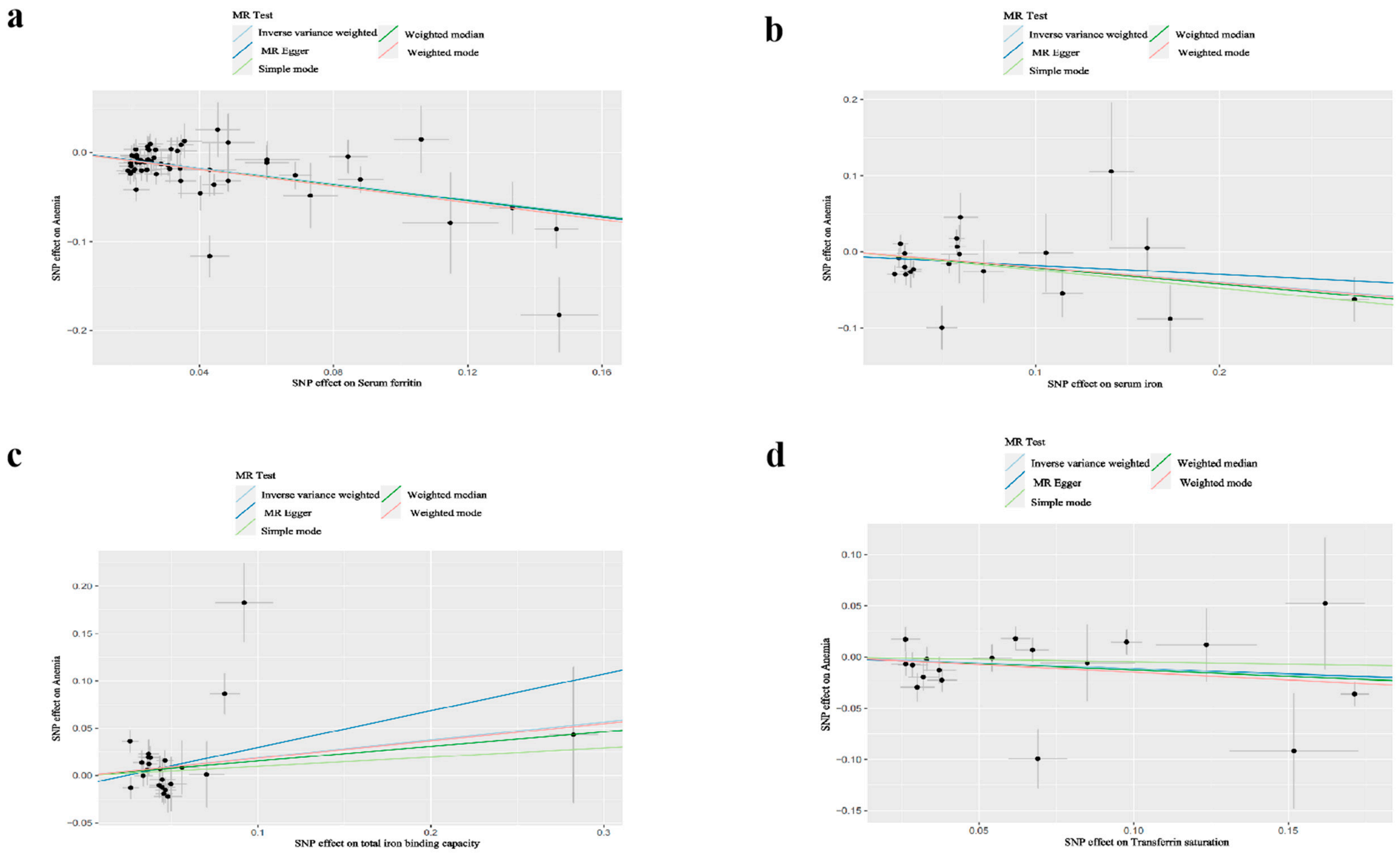

Fig. S3 Scatter plot of the causal association between serum iron status indicators and anemia before removing the outliers. Abbreviations: SNP, Single nucleotide polymorphism; MR, Mendelian Randomization.

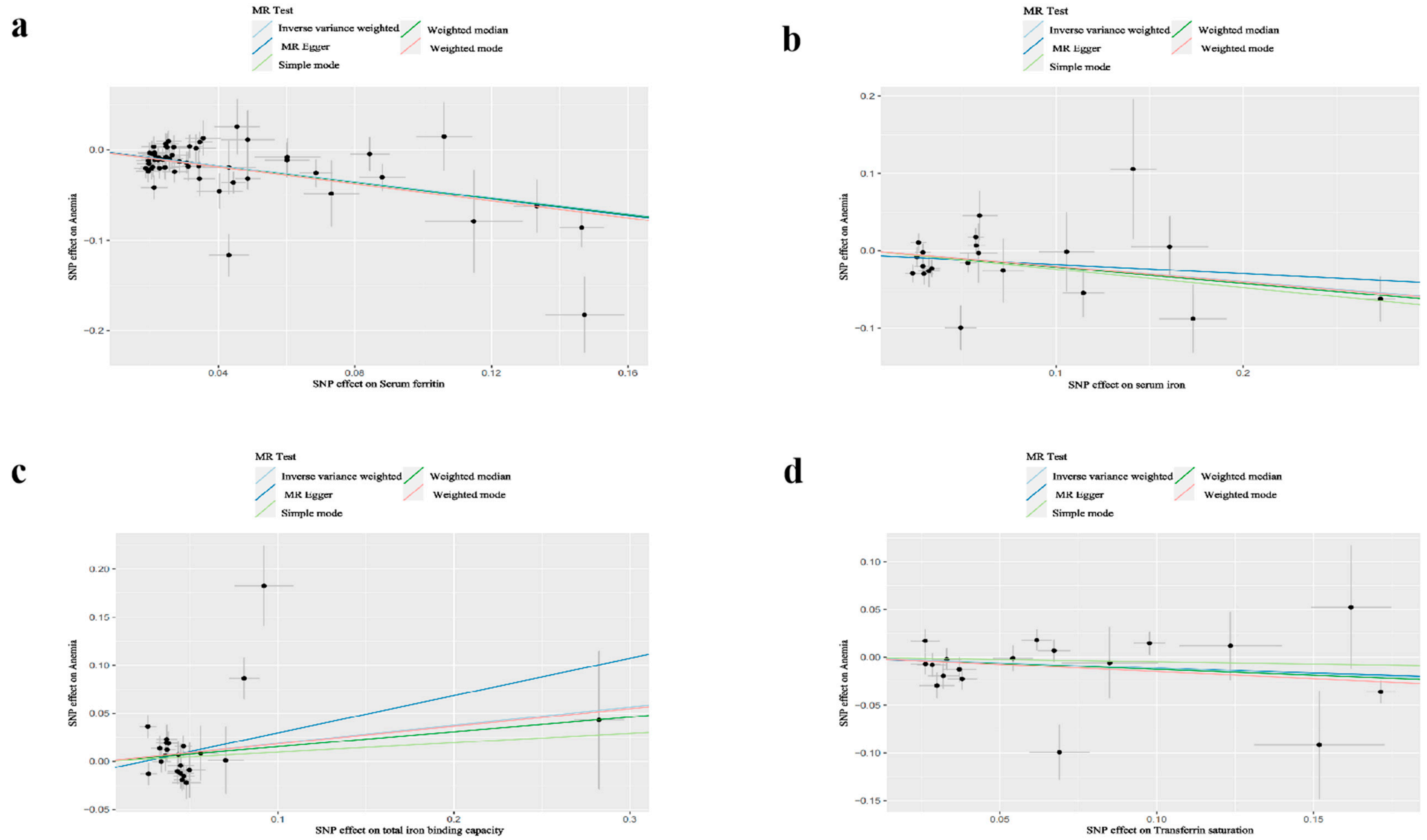

Fig. S4 Scatter plot of the causal association between serum iron status indicators and anemia after removing the outliers. Abbreviations: SNP, Single nucleotide polymorphism; MR, Mendelian Randomization.

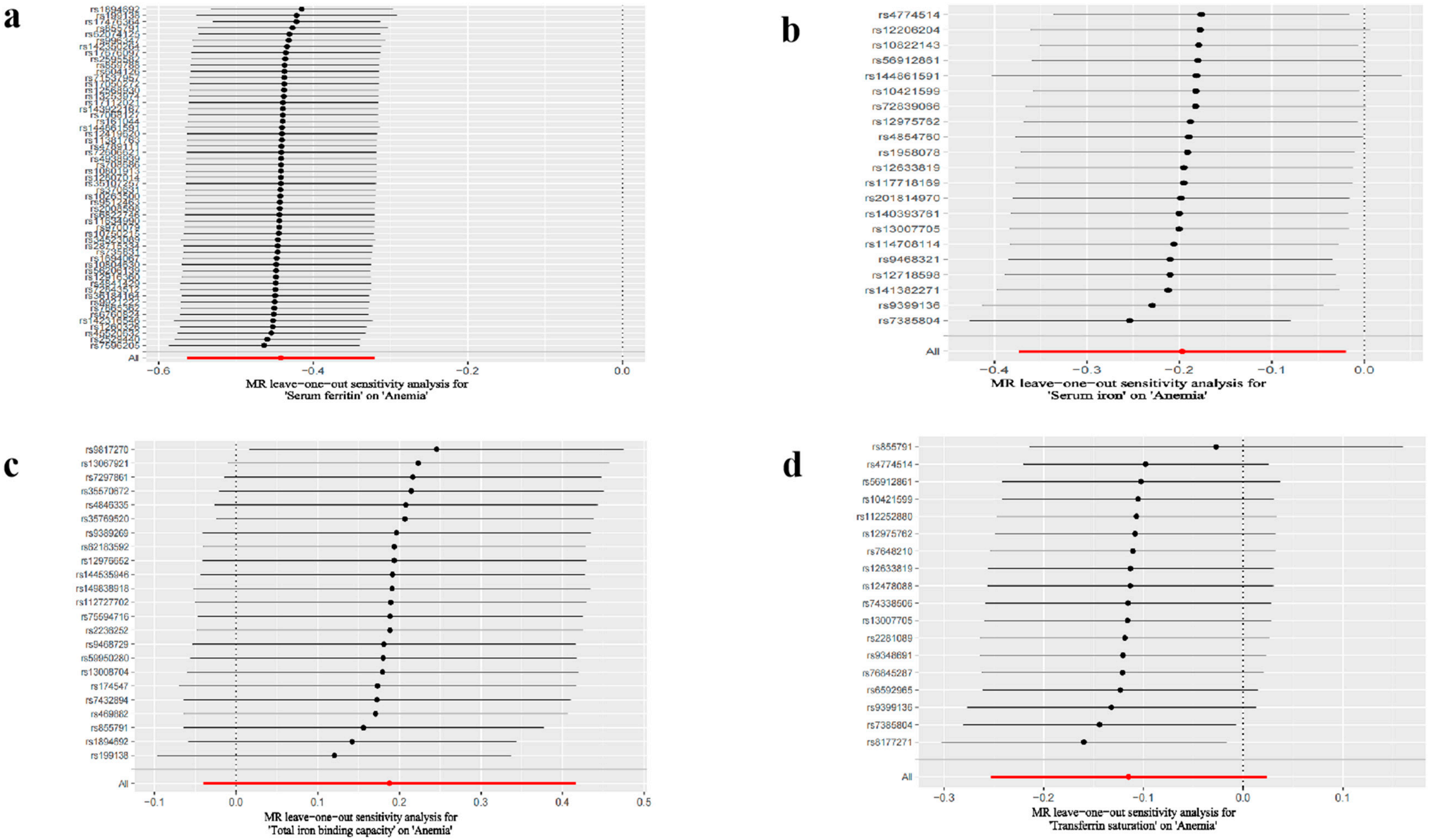

Fig. S5 Leave-one-out analysis for the causal association between serum iron status indicators and anemia before removing the outliers. Abbreviations: MR, Mendelian Randomization.

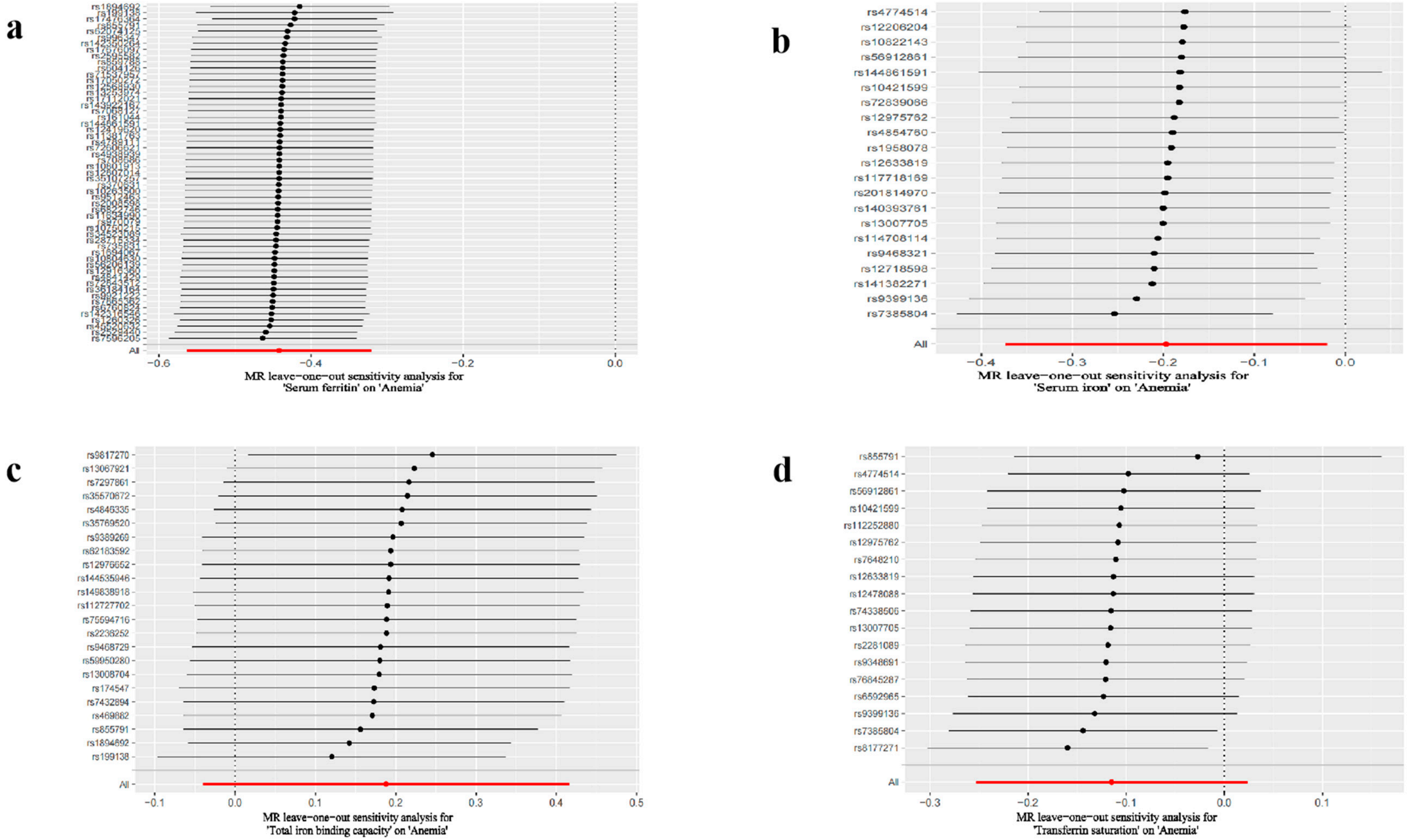

Fig. S6 Leave-one-out analysis for the causal association between serum iron status indicators and anemia after removing the outliers. Abbreviations: MR, Mendelian Randomization.

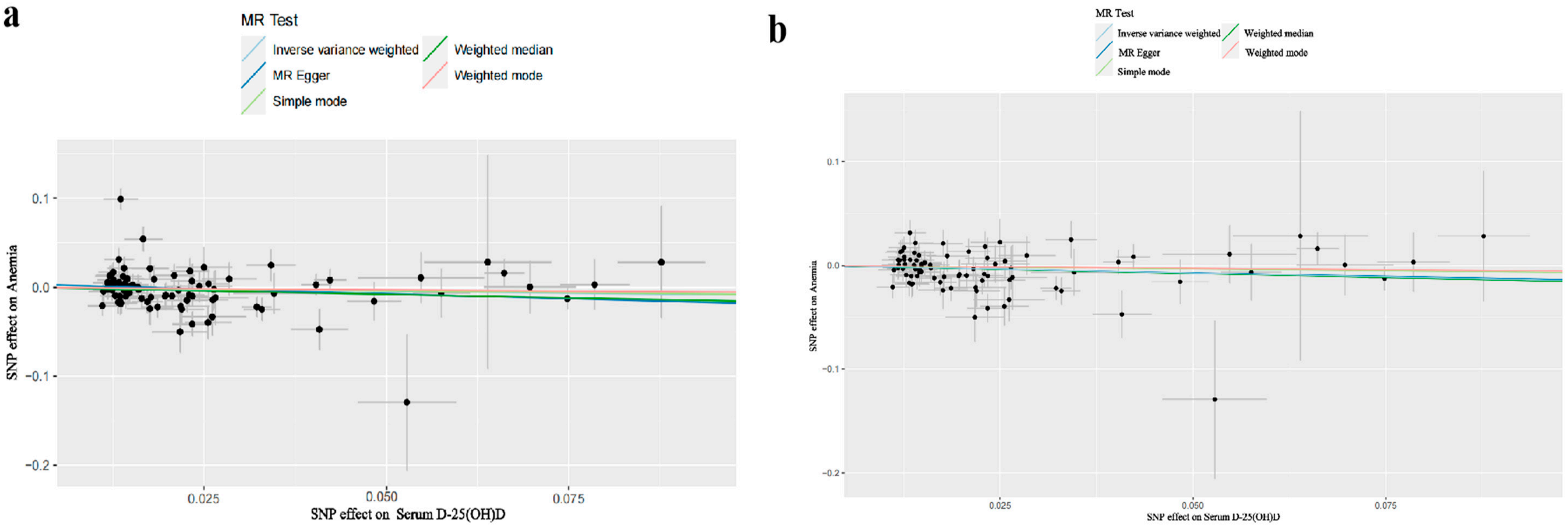

Fig. S7 Scatter plot of the causal association between serum 25-Hydroxyvitamin D and anemia. Abbreviations: SNP, Single nucleotide polymorphism; MR, Mendelian Randomization.

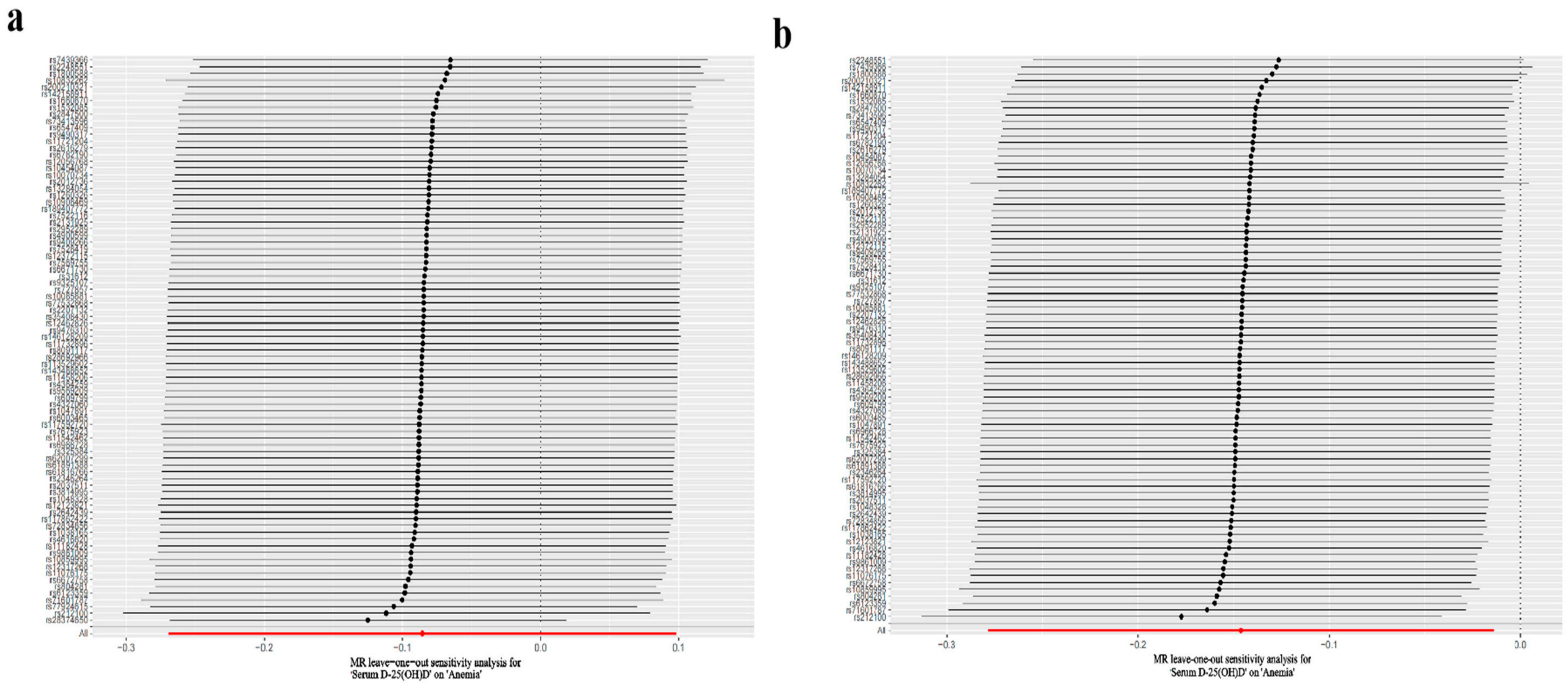

Fig. S8 Leave-one-out analysis for the causal association between serum 25-Hydroxyvitamin D and anemia. Abbreviations: MR, Mendelian Randomization.

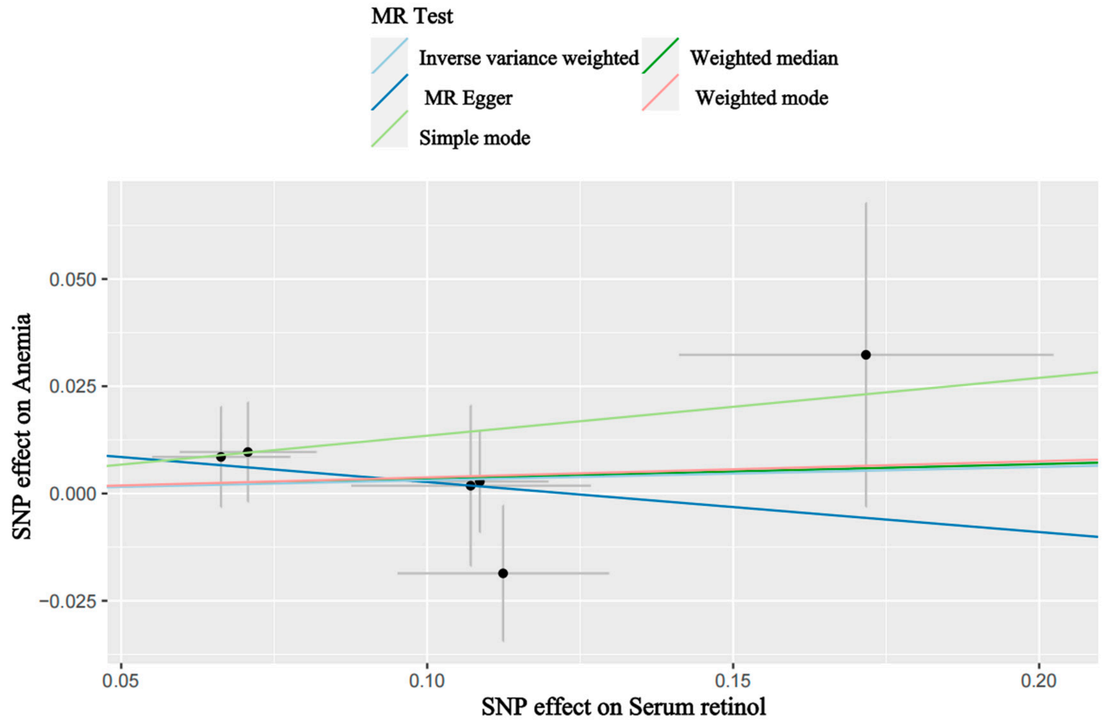

Fig. S9 Scatter plot of the causal association between serum retinol content and anemia. Abbreviations: SNP, Single nucleotide polymorphism; MR, Mendelian Randomization.

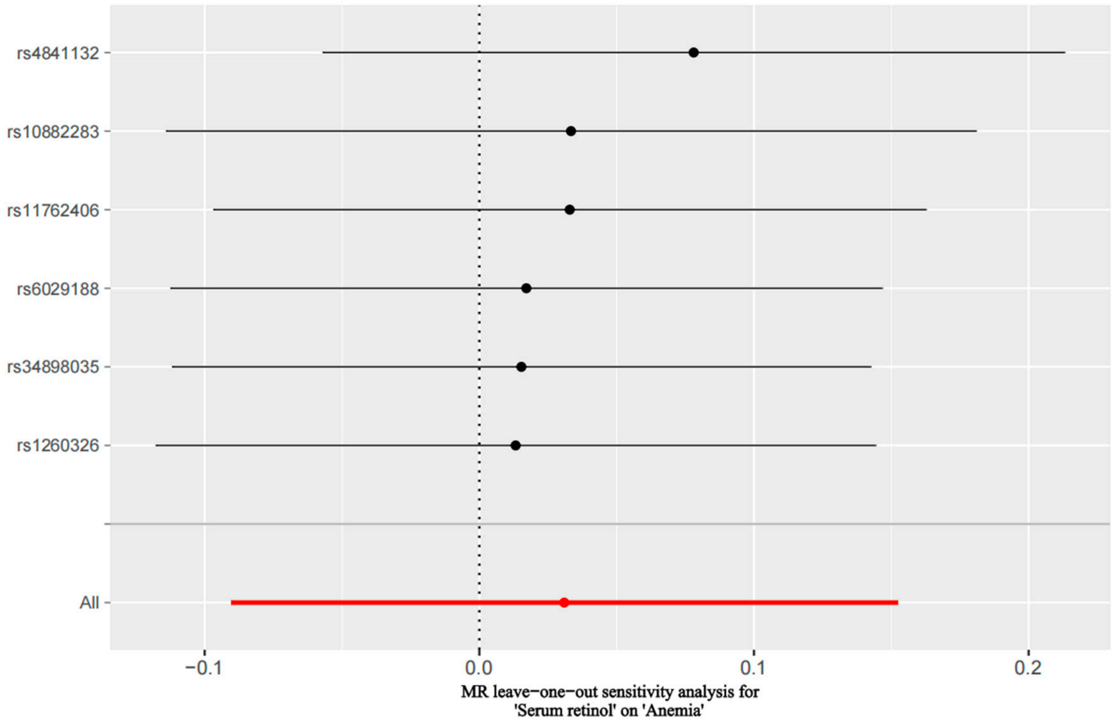

Fig. S10 Leave-one-out analysis for the causal association between serum retinol content and anemia. Abbreviations: MR, Mendelian Randomization.

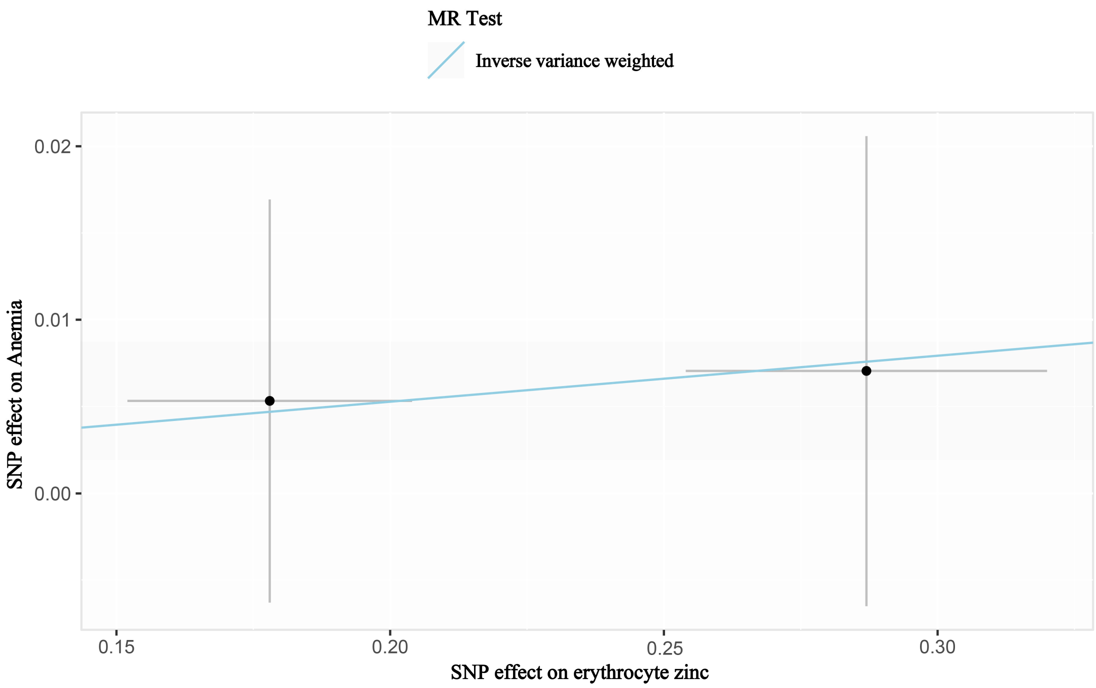

Fig. S11 Scatter plot of the causal association between erythrocyte zinc and anemia.

Abbreviations: SNP, Single nucleotide polymorphism; MR, Mendelian Randomization.
